# *Astragali Radix–Curcumae Rhizoma* synergistically potentiates 5-fluorouracil antitumor efficacy in colorectal cancer by disrupting DNA repair-cell cycle coordination

**DOI:** 10.1186/s13020-026-01449-2

**Published:** 2026-10-21

**Authors:** Shasha Wang, Kexuan Huang, Yuhan Cheng, Cheng Sun, Kan Wang, Qirui He, Gang Yin, Decai Tang, Yu Zhang, Xiying Tan

**Affiliations:** 1 Department of Pharmacy, Affiliated Hospital of Nanjing University of Chinese Medicine, Nanjing, 210029 People’s Republic of China; 2 The First Clinical Medical College of Nanjing University of Chinese Medicine, Nanjing, 210023 People’s Republic of China; 3 School of Pharmacy, Nanjing Medical University, Nanjing, 211166 People’s Republic of China; 4 Department of Pharmacy, Sir Run Run Hospital, Nanjing Medical University, Nanjing, 211166 People’s Republic of China; 5 China College of Sports and Health, Nanjing Sport Institute, Nanjing, 210014 People’s Republic of China; 6 School of Chinese Medicine, Nanjing University of Chinese Medicine, Nanjing, 210023 People’s Republic of China

**Keywords:** *Astragali Radix–Curcumae Rhizoma*, 5-Fluorouracil, DNA process, RNA-seq, Synergistic effect

## Abstract

**Background:**

*Astragali Radix* (HQ) and *Curcumae Rhizoma* (EZ), collectively termed AC, are commonly used as an herb pair in Traditional Chinese Medicine (TCM) for colorectal cancer (CRC). Although AC has been used as an adjuvant to chemotherapy, the molecular basis by which it enhances the efficacy of 5-fluorouracil (5-FU) remains incompletely defined.

**Methods:**

The antitumor effect of AC alone and in combination with 5-FU was evaluated in a HCT116 xenograft mouse model and in CRC cell lines with different genetic backgrounds. Potential systemic toxicity was assessed using serum biochemical markers. Transcriptomic changes induced by AC were analyzed by RNA sequencing and integrated with network pharmacology and The Gene Expression Omnibus (GEO) (GSE17536, GSE17537, GSE41258, GSE20916). Key targets were validated by Western blotting and immunofluorescence. Cell viability, drug interaction, apoptosis, cell-cycle distribution and DNA damage were assessed using CCK-8 assays, Synergy Finder analysis, flow cytometry, and γ-H2AX staining. Bioavailable AC-derived constituents were characterized by UPLC-QTOF-MS, and candidate active compounds were further evaluated by molecular docking, molecular dynamics simulation, cellular thermal shift assay, and functional validation.

**Results:**

AC potentiated the antitumor efficacy of 5-FU in vivo and did not exacerbate 5-FU-associated hepatic or renal biochemical toxicity under the tested conditions. In vitro, AC-containing serum extracts inhibited CRC cell viability, induced reversible G2/M-phase arrest, and enhanced 5-FU sensitivity in HCT116, CT26, and HCT15 cells. AC treatment caused limited early apoptosis, whereas delayed apoptosis was observed after prolonged exposure, suggesting that cell-cycle arrest represents the primary early response. RNA-seq and integrative analyses showed that AC suppressed pathways associated with DNA replication, mismatch repair, ribosome biogenesis, and cell-cycle progression. AC downregulated key regulators involved in cell-cycle control, DNA repair, and stress-survival signaling, including PLK1, CDK1, CCNB1, CDC25A, c-JUN, MSH2, BRCA1, and p-AKT. Combination treatment further increased γ-H2AX accumulation and reduced cell-cycle regulatory proteins. Functional validation indicated that c-JUN overexpression partially attenuated AC-mediated 5-FU sensitization, whereas pharmacological AKT inhibition partially mimicked the sensitizing effect of AC. Among the serum-exposed constituents, Demethoxycurcumin and Kumatakenin were identified as bioavailable active compounds that partially recapitulated the effects of AC by inducing G2/M-phase arrest and enhancing the antiproliferative activity of 5-FU. Molecular docking, molecular dynamics simulation, and CETSA supported their target engagement with cell-cycle regulators, particularly CCNB1 and CDK1.

**Conclusion:**

AC enhances the therapeutic efficacy of 5-FU in CRC by weakening DNA repair-cell-cycle coordination and stress-survival signaling, thereby increasing 5-FU-induced DNA damage and promoting cell-cycle arrest. Demethoxycurcumin and Kumatakenin may represent important bioavailable contributors to this chemosensitizing effect, although the complete AC extract likely acts through multi-component and multi-target mechanisms. These findings provide mechanistic support for the use of AC as an adjunctive strategy to improve 5-FU-based chemotherapy in CRC.

**Graphical Abstract:**

**Figure:**
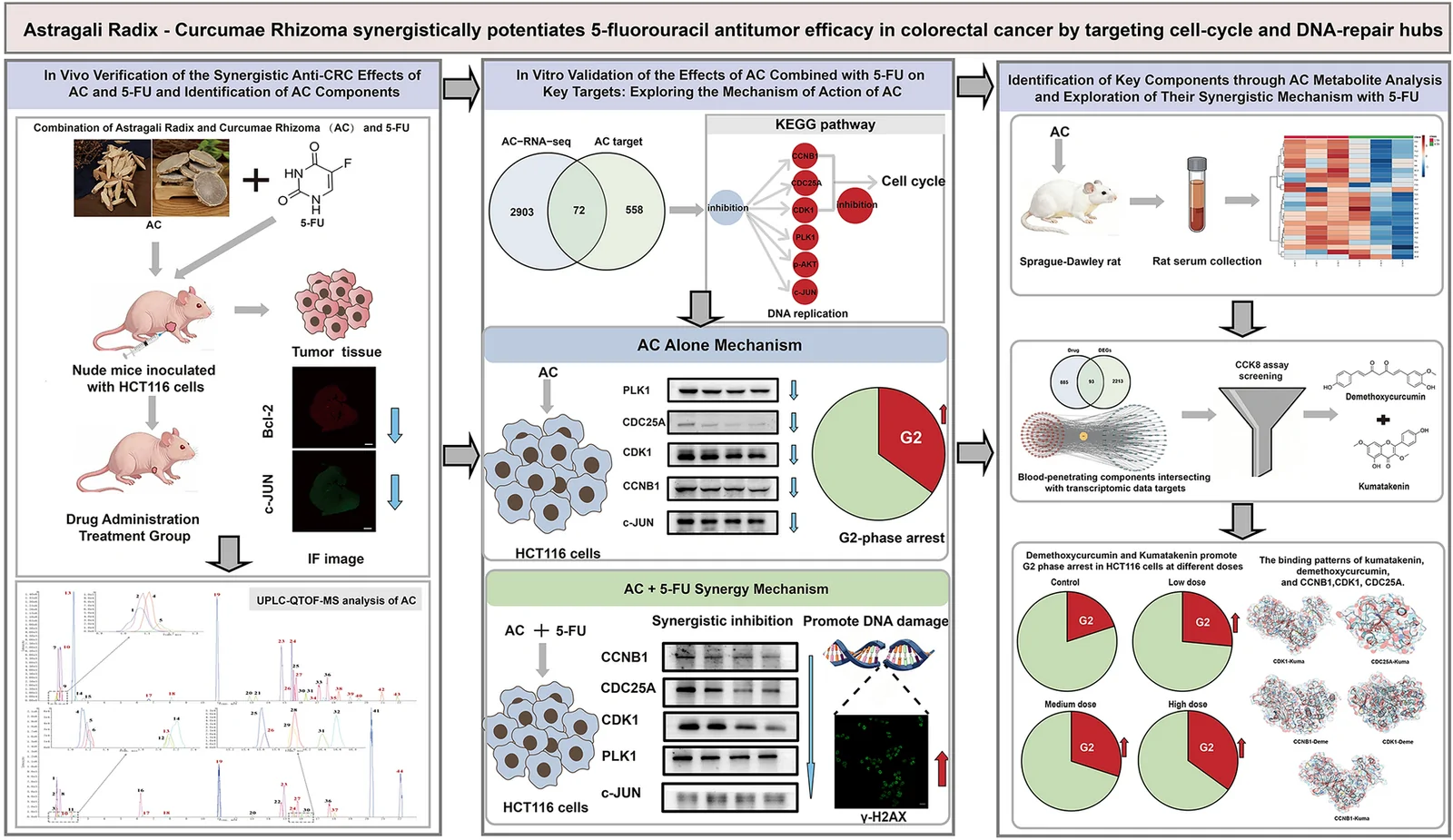

**Supplementary Information:**

The online version contains supplementary material available at https://doi.org/10.1186/s13020-026-01449-2.

## Introduction

Colorectal cancer (CRC) remains a major global health burden. According to Global Cancer Statistics 2022 estimates, approximately 1.9 million new CRC cases and 904,000 CRC-related deaths occurred worldwide, making CRC the third most commonly diagnosed cancer and the second leading cause of cancer-related death [1]. Although surgery, chemotherapy, radiotherapy, targeted therapy, and immunotherapy have improved patient outcomes, therapeutic resistance remains a major barrier to durable disease control and long-term survival.

Fluoropyrimidine-based chemotherapy remains a backbone of CRC treatment. 5-Fluorouracil (5-FU), either as an infusional agent or as part of combination regimens such as FOLFOX and FOLFIRI, is widely used in adjuvant, first-line, second-line, and maintenance treatment settings, particularly for patients with microsatellite-stable or mismatch repair-proficient advanced CRC [2]. Mechanistically, 5-FU is an antimetabolite that is converted into several active metabolites. Among them, 5-fluoro-2'-deoxyuridine-5'-monophosphate (FdUMP) forms a stable ternary complex with thymidylate synthase and 5,10-methylenetetrahydrofolate, thereby suppressing deoxythymidine monophosphate synthesis and interfering with DNA replication and repair [3]. In addition, fluorinated metabolites of 5-FU can be incorporated into RNA and DNA, leading to disrupted RNA processing, defective protein synthesis, nucleotide imbalance, replication stress, and DNA damage [3]. Recent evidence further suggests that an RNA damage response during ribosome biogenesis may represent an important determinant of 5-FU-mediated CRC cell inhibition [4]. These effects activate cell-cycle checkpoints and may trigger programmed cell death, including apoptosis.

The cellular response to 5-FU-induced stress is shaped by DNA damage checkpoints, cell-cycle regulation, and cell-death pathways. The ATR-CHK1 axis primarily coordinates the intra-S phase checkpoint and stabilizes stalled replication forks, whereas the ATM-CHK2 axis is more closely associated with DNA double-strand break responses and p53-dependent damage signaling [5, 6]. Since 5-FU can induce both replication stress and DNA damage in a dose- and context-dependent manner, these checkpoint pathways may jointly determine whether tumor cells repair the damage, arrest the cell cycle, or undergo cell death. Downstream of these checkpoint responses, G2/M transition is regulated by the CDK1-CCNB1 complex, CDC25 phosphatases, and PLK1. Aberrant activation of these regulators may allow CRC cells to maintain proliferation despite chemotherapy-induced stress. Consistently, PLK1 and CDC25A have been implicated in CRC chemoresistance and 5-FU response [7, 8].

In addition to these cell-cycle regulators, c-JUN, a major component of the activator protein-1 transcription factor complex, is an important stress-responsive transcriptional regulator. The JNK/c-JUN/AP-1 axis can modulate proliferation, survival, apoptosis, and chemotherapy response in CRC cells [9]. Therefore, c-JUN may serve as a transcriptional link between chemotherapy-induced stress, cell-cycle adaptation, and apoptotic threshold. Apoptosis is an important downstream outcome when chemotherapy-induced damage cannot be resolved. The intrinsic mitochondrial pathway is largely regulated by the balance between pro-apoptotic proteins such as Bax and anti-apoptotic proteins such as Bcl-2, and dysregulation of BCL-2 family proteins is closely associated with CRC progression and therapy resistance [10]. Thus, combined strategies that aggravate replication stress, disrupt cell-cycle adaptation, and lower the threshold for cell death may represent a rational approach to improving 5-FU-based chemotherapy.

*Astragali Radix* (HQ) and *Curcumae Rhizoma* (EZ), collectively termed AC, are commonly used as an herb pair in traditional Chinese medicine for cancer-related indications, including CRC [11–16]. According to TCM theory, HQ tonifies "Qi", whereas EZ breaks "Yu", both of which are closely related to cancer pathogenesis and treatment. Previous studies have suggested that AC or related formulations may exert anti-CRC effects through pathways such as HIF-2α/β-catenin, Sp1/ZFAS1/miR-153-3p/CCR5, Wnt5/β-catenin, and epithelial-mesenchymal transition-related signaling [17–20]. Nevertheless, the molecular basis by which AC enhances chemotherapy efficacy remains incompletely defined, particularly with respect to DNA replication, DNA repair, cell-cycle regulation, and apoptosis.

In the present study, we investigated the anti-CRC effects of AC alone and in combination with 5-FU using in vivo and in vitro models. By integrating RNA-seq, network pharmacology, GEO analysis, metabolite profiling, molecular docking, cellular thermal shift assay, and functional validation, we aimed to clarify whether AC potentiates 5-FU efficacy by disrupting DNA repair-cell-cycle coordination and stress-responsive survival signaling in CRC.

## Materials and methods

### Reagents

*Astragali Radix* and *Curcumae Rhizoma* were offered by the Jiangsu Province Hospital of TCM and identified by Professor Tulin Lu of Nanjing University of Chinese Medicine as the dried roots of the legume plant *Astragalus membranaceus* (Fisch.) *Bge.var.mongholicus* (Bge.) *Hsiao* and dried rhizomes of *Curcuma wenyujin* Y.H.Chen et C. Ling. Methanol and acetonitrile (HPLC grade) were purchased from Merck (Darmstadt, Germany). Formic acid (MS grade) was purchased from Fluka Sigma Aldrich (Switzerland). Deionized water was prepared using a Milli-Q system (Millipore, Molsheim, France). APCI positive (PN.4460131, Lot A8262) and negative (PN. 4460134, Lot A9032) calibration solutions for the Triple TOF system were obtained from AB Sciex (Massachusetts, USA). Other agents were purchased from Sigma-Aldrich. Microcentrifuge (Thermo Scientific Company, China), WH2 miniature vortex mixer (Shanghai Huxi analytical instrument factory, China), and CPA225D electronic balance (Sartorius Company, Germany) were used for sample preparation. Composed of an Agilent 1290 Infinity Ultra Performance Liquid Chromatography system and a Triple TOF 5600 mass spectrometer (AB SCIEX, United States), the UHPLC-QTOF-MS system was used for sample analysis.

### Preparation of AC extract

*Astragali Radix* and *Curcumae Rhizoma* were mixed at a ratio of 2:1 (w/w) and extracted with 100 mL distilled water for 1 h at 100 ℃. The extract was concentrated to 1 g crude drugs/mL. After cooling to room temperature, the extract was centrifuged at 12,000 × g for 10 min and the supernatant was sterilized by filtration through a 0.22 μm filter. The filtered extract was separated and stored at −20 ℃. To characterize the major chemical components of AC, AC preparations were analyzed by UPLC-QTOF-MS and the typical total ion chromatogram of the AC preparation is presented in Supplementary Fig. 1.

### Animal experiments

#### Collection of rat biological samples for metabolite profiling

Six male Sprague–Dawley rats (200–220 g, SPF grade) were obtained from Beijing Vital River Laboratory Animal Technology Co., Ltd. [Certificate No.: SCXK(Jing)20210006]. After a 3-day acclimation period under controlled conditions (temperature: 22 ± 2 °C; relative humidity: 50 ± 10%; 12 h light/dark cycle) with free access to food and water, rats were randomly divided into a control group and an AC-treated group (n = 3 per group). All animal procedures were conducted in accordance with protocols approved by the Animal Ethics Committee of Nanjing University of Chinese Medicine (Approval No. 2023DW-034-01).

Before administration, all animals were fasted for 12 h with free access to water. Rats in the AC group were orally administered AC aqueous extract at 8.1 g/kg, which was converted from the clinical human dose based on body surface area normalization. Rats in the control group received an equal volume of 0.5% (w/v) sodium carboxymethyl cellulose (CMC-Na). After administration, rats were individually housed in metabolic cages, and urine and fecal samples were collected during 0–6 h, 6–12 h, and 12–24 h intervals. Blood samples were collected from the retro-orbital venous plexus at 1.5 h and 4.5 h after AC administration under anesthesia. Serum was obtained after clotting at room temperature for 30 min followed by centrifugation at 3000 rpm for 10 min at 4 °C. All biological samples were stored at − 80 °C until further analysis.

#### Evaluation of the synergistic effect of AC with 5-FU in vivo

BALB/c Nude Crlj mice: SPF grade, male, 17–19 g, provided by Zhejiang Viton Lihua Laboratory Animal Technology Co., Ltd, Production Licence No.: SCXK (Zhejiang)2019-0001. Animal Quality Certificate No.: 20211103Abzz0619000729. Animal experiments were approved by the ethical committee review of Nanjing Medical University (IACUC-2110014).

HCT116 cells were cultured until the logarithmic growth phase, then rapidly rinsed with trypsin. After discarding the trypsin, the cells were incubated in the incubator for 1 min. Culture medium was then added, and the cells were gently dislodged by pipetting. The cells were centrifuged (4000 rpm, 5 min). The cells were then resuspended in PBS to a concentration of 1.0 × 10⁷ cells/mL and inoculated subcutaneously into the left axilla of nude mice in 100-μL aliquots. The mice were weighed and measured daily for evaluation, and were grouped when the tumors reached approximately 4 mm in size.

Twenty-four tumor-bearing nude mice were randomly divided into four groups (n = 6 per group): model, 5-FU, AC, and AC plus 5-FU groups. Mice in the model group received saline by oral gavage once daily. Mice in the 5-FU group received 5-FU at 30 mg/kg by intraperitoneal injection every other day. Mice in the AC group received AC extract at 3.9 g/kg/day by oral gavage. Mice in the combination group received AC extract at 3.9 g/kg/day by oral gavage and 5-FU at 30 mg/kg by intraperitoneal injection every other day.

The 5-FU dose was selected based on previous CRC xenograft studies using 30 mg/kg 5-FU administered intraperitoneally every other day, together with our pilot tolerability observations [21, 22]. The AC dose was calculated from commonly used clinical doses of *Astragali Radix* and *Curcumae Rhizoma* according to body surface area conversion. Based on the commonly used daily clinical doses of 20 g of Astragalus and 10 g of Curcuma, the total crude drug dose is 30 g/day; calculated for an adult weighing 70 kg, the equivalent adult dose is approximately 0.43 g/kg. Further, using a body surface area conversion factor of 9.1 between adults and mice, the equivalent dose for mice is approximately 3.9 g/kg; and the co-administration group was given 3.9 g/kg/day of AC solution by gavage once a day, and 5-FU solution 30 mg/kg was given by intraperitoneal injection once every other day. Starting from the day of drug administration, the body mass of nude mice was weighed at the same time every day using an electronic balance and recorded, and the tumor volume was calculated by measuring the long diameter (a) and short diameter (b) of the tumor using an electronic vernier caliper on alternate days. Tumor volume (mm^3^) = ab^2^/2.

Seven days after drug administration, mice were anesthetized via inhalation anesthesia, mice were placed in an induction chamber and anesthetized with 4–5% isoflurane. Once respiratory and circulatory functions stabilized and reflexes were suppressed, decapitation was performed. Immediately, the subcutaneously transplanted tumors were dissected and quickly and safely removed, cleaned and blotted in PBS at 4 ℃, and then placed on an electronic precision balance to weigh the mass of transplanted tumors in each group and calculate the mean value of each group to derive the rate of tumor inhibition. The tumors were cut into two parts, one of which was immersed in 4% paraformaldehyde for fixation, and the other was stored in an ultra-low temperature refrigerator at −80 ℃ for subsequent experiments.

### Sample preparation

#### Preparation of serum samples

The collection time points and conditions for serum, urine, and fecal samples are described in Sect. 2.3.1. All collected samples were stored at − 80 °C before preparation.

After thorough vortex mixing, an aliquot (200 μL) of serum was transferred into a microcentrifuge tube and combined with 400 μL of methanol. The mixture was vortexed vigorously and then centrifuged (12,000 × g, 10 min, 4 °C) to pellet protein precipitates. The resulting supernatant was collected.

To further remove residual proteins, 400 μL of methanol was added to 400 μL of the collected supernatant. The mixture was vortexed and centrifuged again under the same conditions (12,000 × g, 10 min, 4 °C). Subsequently, 600 μL of the clarified supernatant was transferred and evaporated to dryness under reduced pressure at 40 °C. The dried residue was reconstituted in 100 μL of 50% aqueous methanol (v/v) with vortex assistance. Finally, the solution was centrifuged (12,000 × g, 10 min, 4 °C), and 60 μL of the supernatant was collected for subsequent UPLC-Q-TOF-MS analysis. All samples were processed in parallel under identical conditions. The collected serum samples were used for subsequent cell-based experimental validation.

#### Preparation of urine samples

Urine samples were thawed at room temperature and vortexed thoroughly. To remove particulate matter, the samples were centrifuged (12,000 × g, 10 min, 4 °C), and the supernatant was collected for subsequent solid-phase extraction (SPE). A 600 μL aliquot of the clarified urine was loaded onto an SPE column that had been preconditioned sequentially with 3 mL of methanol and 3 mL of ultrapure water. Following sample loading, the column was washed with 2 mL of water to remove impurities. The cartridge was then dried thoroughly under vacuum, and the target analytes were eluted with 1.8 mL of methanol. The eluate was collected and evaporated to dryness under reduced pressure at 40 °C. The resulting residue was reconstituted in 600 μL of 50% aqueous methanol (v/v) with vortex mixing. Finally, the reconstituted solution was centrifuged (12,000 × g, 10 min, 4 °C), and 200 μL of the supernatant was collected for UPLC-Q-TOF-MS analysis.

#### Preparation of feces samples

Approximately 0.2 g of each fecal sample was weighed and homogenized with 1 mL of 50% aqueous methanol (v/v) by vortex mixing for 5 min, followed by sonication for 30 min. From the resulting suspension, a 400 μL aliquot was transferred and diluted with 3.6 mL of water. After thorough mixing, the mixture was centrifuged at 4000 × g for 10 min. Subsequently, 3 mL of the supernatant was subjected to solid-phase extraction (SPE) under the same conditions as described for urine samples (preconditioning, washing, and elution). The collected eluate was evaporated to dryness under reduced pressure at 40 °C. The residue was reconstituted in 600 µL of 50% aqueous methanol (v/v) with vortex assistance. Finally, the solution was centrifuged at 12,000 × g (4 °C, 10 min), and 200 µL of the supernatant was collected for UPLC-Q-TOF-MS analysis.

### Chromatography and mass spectrometry analysis

The UPLC separation was performed on an Agilent 1290 Infinity Ultra Performance Liquid Chromatography system equipped with a binary pump (G4220A), a column Oven (G1316C), an autosampler (G4226A) and a temperature control module for the automatic sampler (G1330B), using a Waters ACQUITY UPLC^®^ HSS T3 column (2.1 × 100 mm, 1.8 μm). The mobile phase consisted of water containing 0.1% formic acid (A) and acetonitrile containing 0.1% formic acid (B), with a gradient program as follows: 0–3.5 min: 99% A; 3.5–8 min: 99–80% A; 8–10 min: 80–78% A; 10–13 min: 78–75% A; 13–14 min: 75–60% A; 14–22 min: 60–35% A; 22–25 min: 35% A; 25–26 min: 35–99% A; 26–31 min: 99% A; the flow rate was 0.4 mL/min and the column temperature was maintained at 30 °C. The injection volume was 5 µL and the autosampler temperature was 4 °C. Mass detection was performed on the Triple TOFTM 5600 (AB SCIEX, United States), a hybrid triple Q-TOF mass spectrometer equipped with an ESI source. Both positive and negative ion modes were employed to provide the abundant adduct ions for validating the presence of each potential compound, as well as the fragment ions for structural characterization. Mass spectrometry automatic calibration delivery system (AB SCIEX, United States) was employed to auto calibrate the mass accuracy of the detection per 2 h, by using APCI positive and negative calibration solutions for the AB SCIEX Triple TOFTM system. All the operations and acquisitions were controlled by Analyst TF 1.6 software (AB SCIEX, United States).

### Strategy of metabolites identification

Based on the raw data obtained from UPLC-QTOF-MS, a metabolites identification strategy for AC in rat serum, urine and feces was established with systematic data processing and screening. Firstly, the possible metabolic pathways of various types of compounds in AC were collected and summarized by searching the literatures on metabolites related to AC. A self-built metabolites library of AC was constructed in combination with the compounds in AC and collected metabolic pathways, which contained information of metabolites such as name, molecular formula, and mass-to-charge ratio (m/z). Then, the mass spectrometry data of AC was loaded into MS-DIAL 4.92 software to be analyzed with the self-built metabolites library. Thirdly, the features were optimized based on RT and m/z by MS-FLO website (https://msflo.fiehnlab.ucdavis.edu/), which were extracted through MS-DIAL 4.92 software. Next, the optimized peak intensities data was analyzed using the “Statistical Analysis [one factor]” function of the MetaboAnalyst 6.0 website (https://www.metaboanalyst.ca). The features with fold change threshold (FC) greater than 5 and peak intensity higher than 500 cps were screened and retained as possible AC metabolites. Finally, the raw data obtained by UPLC-QTOF-MS was loaded in PeakView 2.2 software and the RT and m/z of the screened features were imported to extract ion peaks with XIC Manage, whose integrity was confirmed. And two or more adducts of the corresponding features were ensured. Subsequently, the secondary mass spectrometry information of the features was matched and compared with that of chemical constituents in AC. The connections between them were observed to preliminarily infer the structure and identify the metabolites.

The identified serum, urine and feces metabolites of AC were statistically analyzed using MetaboAnalyst 6.0 website. Partial least squares-discriminant analysis (PLS-DA) was used to perform multivariate statistical analysis of serum, urine and feces metabolites to observe the connection of metabolites between different groups. Meanwhile, heatmap was used to compare the differences in the content of various metabolites in different groups.

### Cell culture

HCT116 cells were purchased from FuHeng Biology (Shanghai, China) and cultured in complete medium (McCoy’s 5A (Gibco, USA) containing 10% FBS (Bioind, Kibbutz Beit-Haemek, Israel), 100 μg/mL ampicillin, and 100 μg/mL streptomycin) at 37 ℃ with 5% CO_2_ and 95% air. 5-FU was purchased from Solarbio (IF0170, Beijing, China).

Serum extracts from AC-treated animals and control animals were prepared as described in Sect. 2.4.1. The dried residues were dissolved in dimethyl sulfoxide (DMSO) and diluted with culture medium before use. The final concentration of dimethyl sulfoxide (DMSO) was 0.025% (v/v) in all groups (including the control group).

### Cell viability assay

HCT116 cells were seeded into 96-well plate at a density of 8 × 10^3^ cells/well and treated with AC-containing serum extracts, Demethoxycurcumin (aladdin, 22608-11-3) and Kumatakenin (MCE, 3301-49-3) at the indicated concentrations and time points. Cell viability was assessed by the CCK-8 assay according to the user manual. Briefly, 10 μL/well CCK-8 (APExBIO, USA) was added to the 96-well plates and cultured for another 1 h, then the optical density (OD) was measured at 450 nm on a multifunction microplate reader (Infinite F50, Tecan, Swiss). The proliferation inhibition rate was calculated as per the following equation: (1-OD treated/OD control) × 100%.

### RNA-seq analyses

Total RNA was extracted using the mirVana miRNA Isolation Kit (Ambion) following the manufacturer’s protocol. RNA integrity was evaluated using the Agilent 2100 Bioanalyzer (Agilent Technologies, Santa Clara, CA, USA). The samples with RNA Integrity Number (RIN) ≥ 7 were subjected to the subsequent analysis. The libraries were constructed using TruSeq Stranded mRNA LTSample Prep Kit (Illumina, San Diego, CA, USA) according to the manufacturer’s instructions. Then these libraries were sequenced on the Illumina sequencing platform (HiSeqTM 2500) by OE Biotech Co., Ltd. (Shanghai, China), and 125 bp/150 bp paired-end reads were generated.

The transcriptome sequencing and analysis were conducted by OE Biotech Co., Ltd. (Shanghai, China). Raw data (raw reads) were processed using Trimmomatic. The reads containing poly-N and the low-quality reads were removed to obtain the clean reads. Then the clean reads were mapped to the reference genome using HISAT2. FPKM value of each gene was calculated using Cufflinks, and the read counts of each gene were obtained by HTSeq-count. Differentially expressed genes (DEGs) were identified using the DESeq (2012) R package functions estimateSizeFactors and nbinomTest. P < 0.05 and |foldchange|> 1 was set as the threshold for significantly differential expression.

### GO and KEGG pathway enrichment analysis

Hierarchical cluster analysis of DEGs was performed to explore gene expression patterns. Gene Ontology (GO) enrichment is used to define and describe the functions of gene products from three aspects, namely, biological processes (BPs), cell components (CCs), and molecular functions (MFs). Kyoto Encyclopedia of Genes and Genomes (KEGG) is a database that integrates genome, chemistry, and system function information. GO and KEGG functional enrichment analysis were performed by the clusterProfiler software package of the R platform with the screening criterion was P < 0.05.

### Western blot

For protein extraction, cells cultured in 12-well plates were treated with drugs for 24 h. The culture medium was then discarded, and the cells were gently rinsed once with phosphate-buffered saline (PBS). After removing the PBS, 200 μL of loading buffer was added to each well. The extracted proteins were separated by 10% sodium dodecyl sulfate–polyacrylamide gel electrophoresis (SDS-PAGE) and subsequently transferred onto polyvinylidene fluoride (PVDF) membranes. The membranes were incubated with primary antibodies at 4 °C for 12 h.

The primary antibodies were as follows: rabbit anti-PLK1 (1:1000, JingTing, JTT6202), mouse anti-Bax (1:1000, proteintech, 60267-1-1 g), rabbit anti-CCNB1 (1:1000, JingTing, JTT8830), rabbit anti-CDC25A (1:1000, JingTing, JTT9203), rabbit anti-c-JUN (1:1000, JingTing, 24909-1-AP), rabbit anti-CDK1 (1:1000, JingTing, JTT9170), rabbit anti-p-AKT (1:1000, CST,4060S), and anti-Bcl-2 (1:1000, CST, 3498T). An internal control was performed using mouse anti-β-actin (1:2000, proteintech, 6600-1-AP), and appropriate horseradish peroxidase-linked secondary antibodies were used for enhanced chemiluminescence detection (Pierce).

### Flow cytometry

For cell cycle analysis, log-phase HCT116 cells were seeded in 6-well plates at a density of 8 × 10^4^ cells/mL. After 8 h, the cells were treated with 1, 2, and 4 μM of Demethoxycurcumin or with 2, 4, and 8 μM of Kumatakenin, respectively. At 24 h post-treatment, the culture medium was gently removed, and the cells were washed once with 1 mL of PBS. After discarding the PBS, the cells were digested with 500 μL of trypsin–EDTA. The digestion was terminated by adding complete culture medium, and the entire cell suspension was gently transferred to centrifuge tubes and centrifuged at 1,500 rpm for 5 min to pellet the cells. The supernatant was discarded, and 1 mL of 1 × propidium iodide (PI) staining solution (5 μg/mL) was added to each test tube, followed by 5 μL of 1 × permeabilization solution. The cells were gently resuspended by pipetting and incubated in the dark at 37 °C for 30 min. Detected by flow cytometry at an excitation wavelength of 488 nm, with simultaneous measurement of light scattering. Cell cycle distribution was analyzed using FlowJo software v10.8.1 For apoptosis analysis, log-phase HCT116 cells were seeded in 6-well plates at a density of 8 × 10^4^ cells/mL. After 8 h, the cells were treated with 1, 2, and 4 μM of Demethoxycurcumin or with 2, 4, and 8 μM of Kumatakenin, respectively. At 24 h post-treatment, the culture medium was gently removed, and the cells were digested with EDTA-free trypsin. The cell suspension was centrifuged at 1,800 rpm for 5 min at 4 °C, and the supernatant was discarded. The cells were washed twice with pre-chilled PBS, each wash followed by centrifugation at 1,800 rpm for 5 min at 4 °C. Then, 100 μL of 1 × binding buffer was added, and the cells were gently pipetted to form a single-cell suspension. Subsequently, 5 μL of Annexin V-FITC and 5 μL of PI staining solution were added, mixed gently, and the mixture was incubated at room temperature in the dark for 10 min. Finally, 400 μL of 1 × binding buffer was added and mixed gently. The stained samples were analyzed by flow cytometry within 1 h.

### Immunofluorescence (IF) assays

For tumor tissue immunofluorescence, tumor samples were fixed in 4% paraformaldehyde, embedded in paraffin, and sectioned at a thickness of 3 μm. After deparaffinization, antigen retrieval, permeabilization, and blocking, sections were incubated overnight at 4 °C in primary antibodies. The sections were then incubated with species-appropriate fluorescent secondary antibodies (Servicebio), followed by nuclear counterstaining with 4′,6-diamidino-2-phenylindole (DAPI). Images were acquired using a fluorescence microscope (Leica DMi8 M/C/A), and fluorescence intensity was quantified using ImageJ software 1.52i.

The primary antibodies were as follows: rabbit anti-CDK1 (1:300, JingTing), rabbit anti- CCNB1 (1:300, JingTing), rabbit anti- PLK1 (1:300, proteintech), rabbit anti- CDC25A (1:300, JingTing), rabbit anti- JUN (1:300, Jingting), rabbit anti- Bcl-2 (1:300, proteintech).

For cellular immunofluorescence, treated cells were fixed with 4% PFA, permeabilized, blocked, and incubated overnight at 4 °C with primary antibodies. After washing with PBS, cells were incubated with species-specific fluorescent secondary antibodies at room temperature in the dark for 2 h. After DAPI counterstaining, images were acquired under a fluorescence microscope at the indicated excitation wavelengths. Quantitative analysis was performed using ImageJ software 1.52i.

### Identifying key compounds and core targets through network pharmacology screening

A total of 140 compounds were collected from TCMSP (https://tcmsp.91medicine.cn) and BATMAN (http://bionet.ncpsb.org.cn/batman-tcm). These compounds were screened using Swiss ADME (www.swissadme.ch), resulting in 102 compounds. Target prediction for these 140 compounds was performed using Swiss Target Prediction (www.swisstargetprediction.ch), yielding 978 predicted targets. The intersection of these 978 targets with 2306 DEGs from the transcriptome data was calculated, resulting in 93 intersecting targets. Subsequently, network pharmacology analysis was performed using Cytoscape 3.10.3 to map compounds to the intersection targets. Compounds were ranked based on their degree values, and the top ten compounds were selected as core compounds. The 93 intersection targets were imported into STRING (https://cn.string-db.org) for analysis. The results were then imported into Cytoscape 3.10.3 and screened using Cytohubba to identify key targets.

### Assessment of drug synergy using SynergyFinder

HCT116 cells were seeded into 96-well plates at a density of 8 × 10^3^ cells/well. After 8 h of attachment, cells were treated with Demethoxycurcumin, Kumatakenin, and 5-FU either alone or in combination at concentrations covering their respective IC₅₀ ranges. After 24 h, 10 μL of CCK-8 reagent (APExBIO, USA) was added to each well, and cell viability was measured as described above. Final concentrations of Demethoxycurcumin were 1, 2, and 4 μM, while Kumatakenin concentrations were 2, 4, and 8 μM. The maximum combination concentration for 5-FU was 10 μM. Drug interactions were analyzed using Synergy Finder software 3.0 based on the zero interaction potency (ZIP) model. A ZIP Synergy score > 10 was considered synergistic effects, whereas a score < −10 was considered antagonistic effects. Corresponding 2D synergy maps were generated to visualize the interaction patterns.

### Molecular docking and molecular dynamics simulation

Molecular docking was performed using Maestro (Schrodinger 2021) software. Proteins were processed via the Protein Preparation Wizard workflow, while ligands were prepared in the LigPrep module. Docking employed the ligand docking mode with a standard precision (SP) model and default parameters. PyMOL was utilized for visualization.

Potential activator-target complex dimensional stability was assessed via 100-ns molecular dynamics simulations using Maestro's Desmond module. The solvation system was predefined with SPC (simple point charge), featuring a 10 × 10 × 10 Å calculation box and supplemented with 0.15 M Na⁺ and Cl⁻ ions. The OPLS4 (optimized potentials for liquid simulations 4) force field was employed. NPT (constant number of particles, pressure, and temperature) simulations were performed at 300 K and 1.01325 bar pressure.

### Cellular thermal shift assay (CETSA)

HCT116 cells were treated with radioimmunoprecipitation assay (RIPA) lysis buffer (P0013C) for 30 min, and the lysates were centrifuged to collect the supernatant. The supernatant was then divided into three groups and treated with 4 μM Demethoxycurcumin, 8 μM Kumatakenin, or DMSO (control, the concentration is expressed as 0.025% by volume), respectively. The extracted supernatants were further aliquoted into eight portions (100 μL per tube). Each portion was heated at a different temperature (input, 35 °C, 40 °C, 45 °C, 50 °C, 55 °C, 60 °C, and 65 °C) for 40 min, followed by centrifugation at 12,000 rpm for 20 min. The resulting supernatant was mixed with loading buffer, heated at 100 °C for 10 min, and subsequently analyzed by Western blotting.

### Statistical analysis

All experiments were independently repeated at least three times, with data expressed as mean ± standard deviation. Statistical analysis was performed using GraphPad Prism 9.5.1 software (GraphPad Software, Inc.). Differences between groups were assessed for statistical significance using one-way analysis of variance (ANOVA) and two-way ANOVA with *p* < 0.05 considered statistically significant.

## Results

### AC synergistically enhances the anti-CRC effect of 5-FU in vivo

**Fig. 1:**
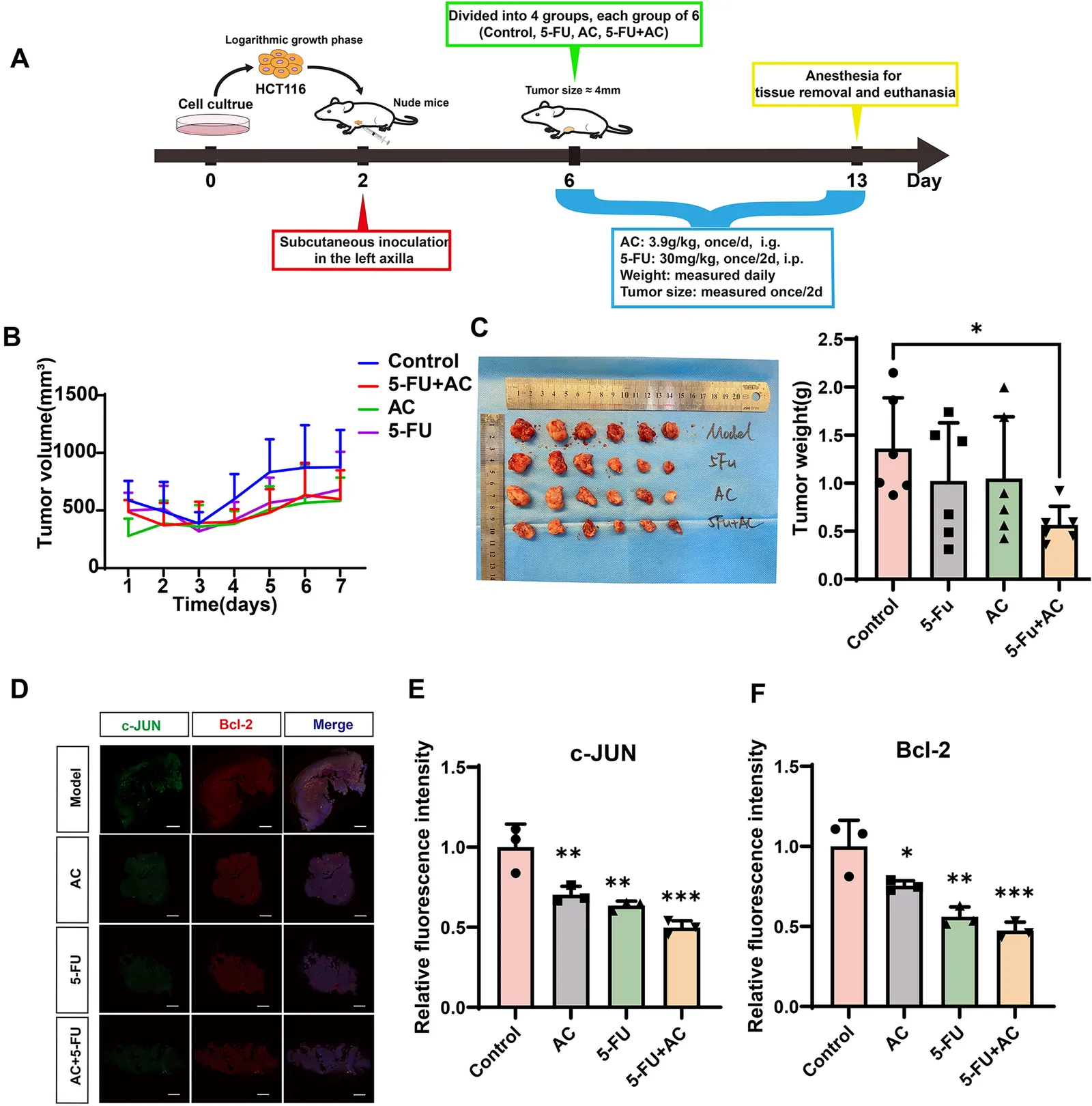
AC significantly increased sensitivity of CRC to 5-FU in vivo. **A** Animal experiment schedule. **B** Statistical graph of changes in tumor volume after treatment **C** Tumor size comparison map after treatment. Tumor weight was statistically analyzed (n = 6). **D**–**F** Immunofluorescence images showing intra-tissue expression levels of c-JUN (green), Bcl-2 (red), and cell nuclei stained with DAPI (blue) (n = 3). (Scale bar: 2000 μm). Data are presented as the mean ± SD. Statistical significance was determined versus the model group (*p < 0.05, **p < 0.01, ***p < 0.001)

To determine whether AC potentiates the antitumor activity of 5-FU in vivo, a HCT116 subcutaneous xenograft model was established in BALB/c nude mice. As shown in Fig. 1A, mice were treated with the saline solution (Control), 5-FU, AC, and 5-FU-AC, in which the rate of HQ-EZ and the dose of AC were selected based on our previous in vivo study [23]. Tumor growth was inhibited by the administration of 5-FU or AC alone as well as in combination compared to the control group (Fig. 1B). The combination treatment exhibited significantly lower final tumor weight and smaller tumor size compared to 5-FU or AC alone (Fig. 1C). We calculated the relevant values based on the final tumor weights in the AC + 5-FU group of mice with colon cancer using the following formula found in the literature (Excess over Bliss score = I = Y_AC 5-FU_-$\widehat{Y}$_AC 5-FU_ $$\left\{\begin{array}{c}>0 Synergism\\ =0 Independent.\\ <0 Antagonism\end{array}\right.$$) [24], and the results showed that $\widehat{Y}$_AC 5-FU_ = 0.420698414, Y_AC 5-FU_ = 0.584873333, and Y_AC 5-FU_ -$\widehat{Y}$_AC 5-FU_ > 0. The above results indicate that AC and 5-FU exhibit a synergistic effect. Subsequently, immunofluorescence analysis of tumor tissues revealed that the combination treatment significantly downregulated the protein levels of the transcription factor c-JUN and the anti-apoptotic protein Bcl-2 compared with either 5-FU or AC alone (Fig. 1D–F). To evaluate the systemic toxicity of the AC + 5-FU combination in mice, we measured serum biochemical parameters, including ALT, AST, BUN, and CREA, in animals from the control group, the 5-FU monotherapy group, and the AC + 5-FU combination group. The results showed that, compared with the 5-FU group, there were no significant differences in ALT, AST, BUN, or creatinine levels in the AC + 5-FU group, suggesting that AC did not exacerbate 5-FU-associated hepatic or renal biochemical toxicity under the present experimental condition (Supplementary Fig. 2). These in vivo data demonstrate that AC enhances the efficacy of 5-FU in CRC.

### AC dose-dependently inhibits HCT116 cell viability and induces cell cycle arrest in vitro

**Fig. 2:**
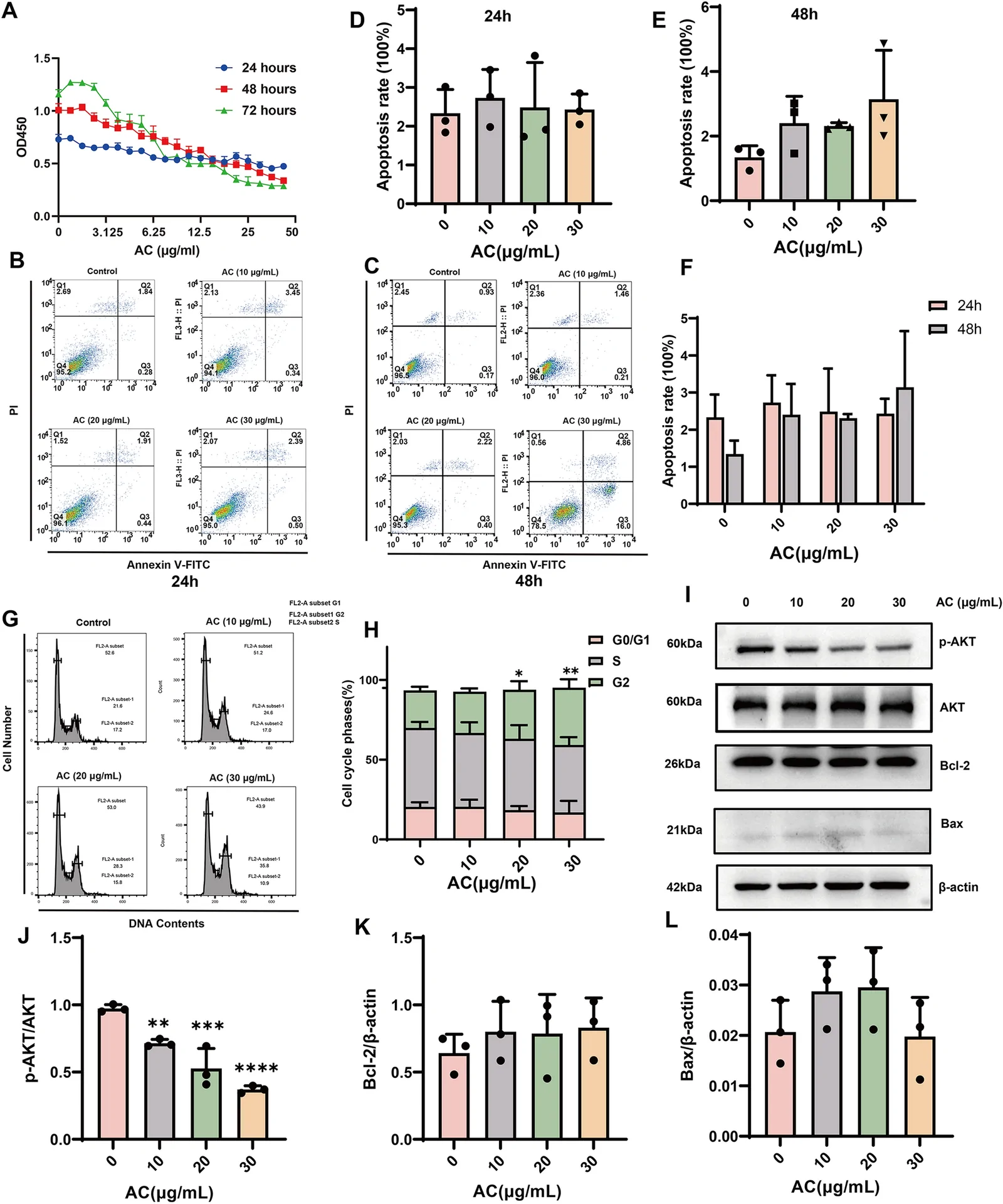
Serum extracts from AC-containing mice inhibited the proliferation of CRC in vitro. **A** CCK-8 assay was used to evaluate the effects of AC-containing serum extracts at different concentrations on HCT116 cells at various treatment time points. **B** Flow cytometry was used to analyze the apoptosis rate in HCT116 cells 24 h after treatment with mouse serum extracts containing varying concentrations of AC. **C** Flow cytometry was used to analyze the apoptosis rate in HCT116 cells 48 h after treatment with mouse serum extracts containing varying concentrations of AC. **D**–**F** Statistical charts and comparison charts showing the apoptosis rates in HCT116 cells after 24-h and 48-h treatment with AC-containing serum extracts at different concentrations. **G**, **H** Cell cycle profiles and statistical analyses of HCT116 cells were performed using flow cytometry 24 h after treatment with AC-containing serum extracts at different concentrations. **I**–**L** Western blot was used to detect p-AKT, total AKT, Bax, and Bcl-2 protein levels. (n = 3) Data are presented as the mean ± SD. Statistical significance was determined versus the model group (**p < 0.01, ***p < 0.001, ****p < 0.0001)

To evaluate the direct anti-CRC effect of AC in vitro, HCT116 cell, a human CRC cell line, was treated with AC-containing serum extracts. CCK-8 assays demonstrated that AC-containing serum extracts inhibited the survival of HCT116 cells in a dose- and time-dependent manner (Fig. 2A). Subsequently, we selected AC-containing serum extracts at concentrations of 10, 20, and 30 μg/mL to further investigate their effects on apoptosis and the cell cycle. Flow cytometry analysis revealed that AC treatment induced cell cycle arrest 24 h later (Fig. 2G, H). To assess whether AC-induced G2/M phase arrest was reversible, the G2/M phase cell proportion in each treatment group was measured 24 h after a 30 μg/mL AC treatment, followed by 24 h of continued culture after discontinuation of the drug. The results showed that the G2/M phase cell proportion in each treatment group had returned to levels close to those of the control group. This indicates that G2/M phase arrest induced by AC and its active components is transient and reversible (Supplementary Fig. 3 A). However, its effect on apoptosis was negligible (Fig. 2B, D). At 24 h, AC treatment induced evident cell-cycle arrest but only minimal apoptosis. To determine whether prolonged arrest was followed by delayed apoptosis, apoptosis was further assessed at 48 h (Fig. 2C, E) and 72 h (Supplementary Fig. 3C, D). Apoptosis increased over time but remained relatively low, even in the high-dose AC group at 72 h, supporting the view that AC primarily induced cell-cycle arrest, with apoptosis occurring as a delayed secondary effect. The expression levels of the anti-apoptotic protein Bcl-2 and the pro-apoptotic protein Bax were examined. Western blot analysis showed that the p-AKT/AKT ratio was reduced in the medium- and high-dose AC groups, whereas Bax and Bcl-2 levels were not markedly altered (Fig. 2I–L).

In conclusion, these results demonstrate that AC directly inhibits the proliferation of HCT116 cells by the modulation of key survival signals to induce cell cycle arrest, rather than the direct activation of apoptosis.

### RNA-seq analysis indicates the suppression of DNA replication, mismatch repair, and cell cycle contributes to the anti-CRC effect of AC

**Fig. 3:**
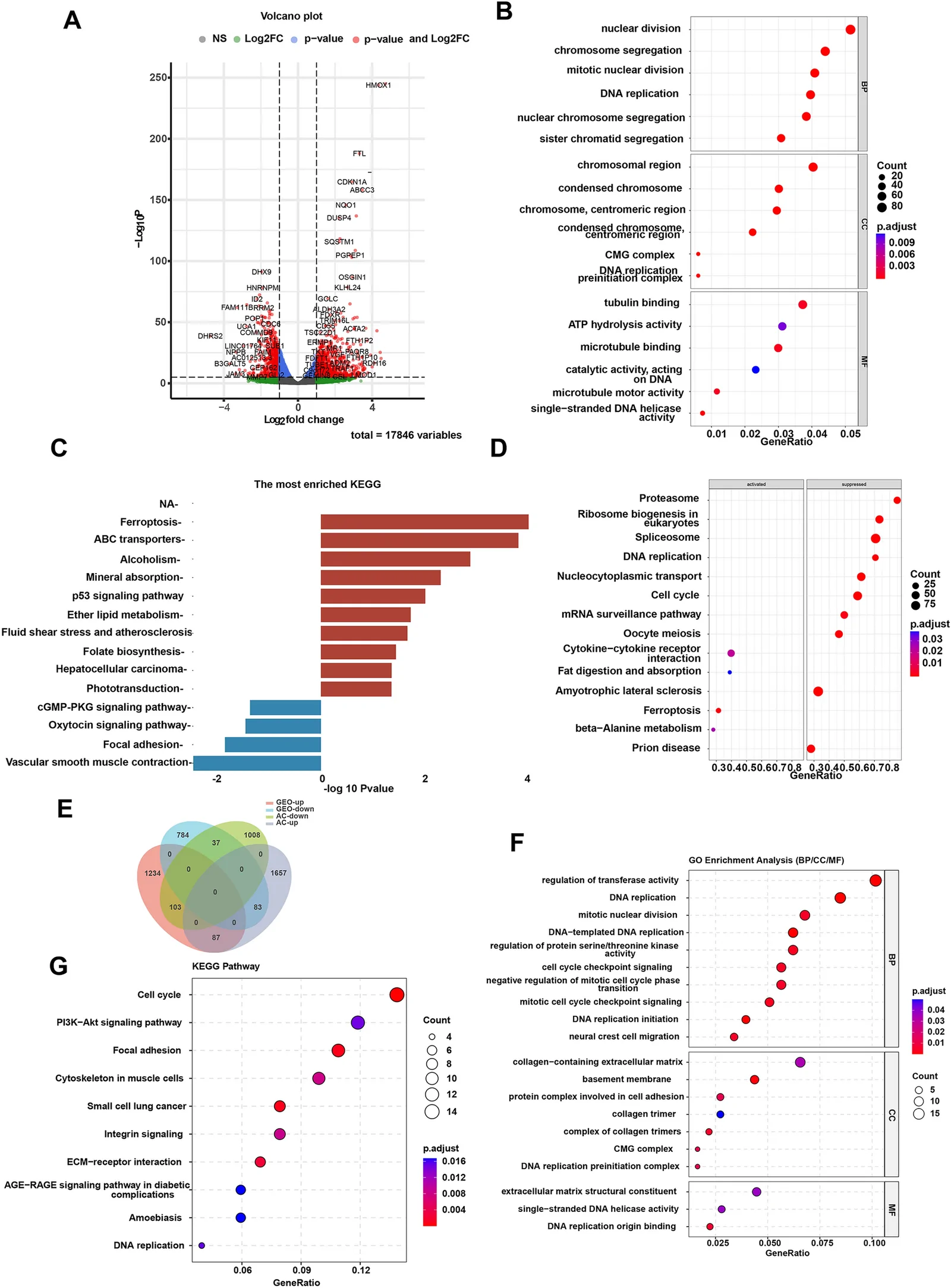
Integration of RNA-seq and GEO explores the mechanism of AC on the inhibition of CRC. **A** Volcano plot showed the administration of AC significantly influenced the profile of gene expressions of HCT116 cell. **B** By GO analysis of DEGs, it indicated that AC influences DNA related processes, such as the replication and many processes related to chromosomes. **C** By KEGG analysis of DEGs, AC significantly increased the damage related signaling pathways, such as ferroptosis and p53 signaling pathways. AC also downregulated the cGMP-PKG signaling pathways and Focal adhesion. **D** GSEA analysis showed that AC significantly suppressed the signaling pathways, such as ribosome biogenesis, splicesome, DNA replication, cell cycle and so on, all of which are tightly related to the cell proliferation. **E** 217 genes downregulated in CRC of GEO database were genes upregulated in the AC group and 355 genes upregulated in CRC of GEO database were genes downregulated in AC group. **F** By GO analysis of common genes between GEO and AC, these genes tightly relate to the DNA and chromosome progress. **G** KEGG analysis of common genes between GEO and AC also shown the signal pathways, such as cell cycle, DNA replication and mismatch repair, were significantly influenced

AC contains many potential active ingredients and these active ingredients can influence various biological behaviors of CRC cells through their targets. RNA-seq reflects the gene expression changes at the transcriptome level; therefore, we evaluated the changes in gene expression following the treatment of HCT116 cells with serum extracts from AC-treated mice or saline-treated mice. As shown in Fig. 3A, 1827 genes were significantly upregulated and 1148 genes were significantly downregulated by the administration of AC. These differentially expressed genes (DEGs) were enriched in several important biological processes, particularly in nuclear division, chromosome segregation, mitotic nuclear division, DNA replication and so on (Fig. 3B). KEGG analysis also showed the upregulation of p53 signaling pathway, Ferroptosis, and other signaling pathways, and the downregulation of signaling pathways relating to genome maintenance and cell-cycle progression (Fig. 3C). GSEA analysis also confirmed the suppression of ribosome biogenesis, cell cycle, spliceosome, and other pathways by the AC administration (Fig. 3D). Interestingly, nucleocytoplasmic transport and mRNA surveillance pathways which involve the stabilization and biogenesis of RNA were also downregulated by AC (Fig. 3D). A comparison of DEGs in patients with CRC from the GEO database with DEGs from our RNA-seq data showed that 355 upregulated genes and 217 downregulated genes in patients with CRC were reversed in their expression by AC treatment (Fig. 3E). The biological functions of these genes were enriched in chromosome segregation, DNA-templated DNA replication, and other processes (Fig. 3F). KEGG analysis of these overlapping genes showed enrichment in pathways related to cell cycle, DNA replication, mismatch repair, and so on (Fig. 3G). These results indicate the influences on genes and pathways relating to DNA replication, repair and stabilization contribute to the beneficial effects of AC.

### Validation of potential targets according to RNA-seq analysis in HCT116 cells in vitro

**Fig. 4:**
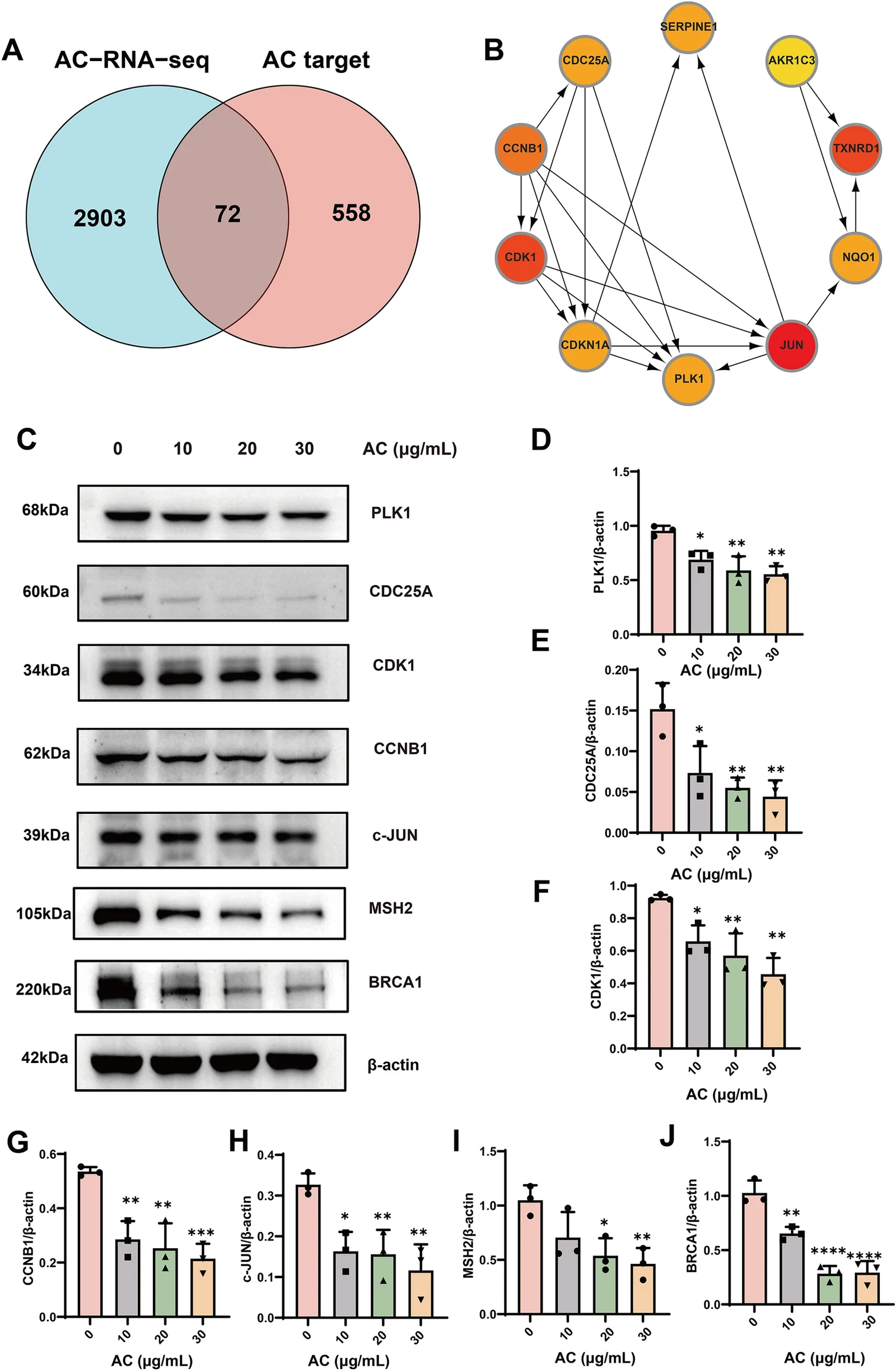
Experimental validations of potential key targets of AC on the inhibition of CRC. **A** Potential key targets of AC according to RNA-seq and network pharmacology. **B** Top 10 targets from PPI analysis **C**–**J** Western blot analysis and quantification of cell-cycle, stress-response-, and DNA repair-related proteins after AC treatment (n = 3). Data are presented as the mean ± SD. Statistical significance was determined versus the model group (*p < 0.05, **p < 0.01, ***p < 0.001 ****p < 0.0001)

Protein–protein interaction (PPI) network analysis of the common genes identified through RNA-seq and network pharmacology found several hub genes, such as Jun Proto-Oncogene (JUN), Cyclin B1 (CCNB1), Cell Division Cycle 25A (CDC25A), and Polo Like Kinase 1 (PLK1), as potential key mediators of anti-CRC effects of AC (Fig. 4A, B). These genes are critically involved in cell proliferation and DNA repair. c-Jun, a key component of the activator protein 1 (AP-1) family, regulates apoptosis and other cellular processes. CCNB1, a cyclin family member, is essential for the G2/M phase transition, and its inhibition decreases proliferative capacity. CDC25A, a phosphatase, primarily regulates the G1-S transition by dephosphorylating CDK complexes. PLK1, a serine/threonine kinase, is a core regulator of cell division, playing important roles in mitosis.

Compared with the control group, the expression levels of proteins associated with cell-cycle regulation, stress-responsive transcription, and DNA repair, including PLK1, CDC25A, CDK1, CCNB1, c-JUN, MSH2, and BRCA1 were significantly reduced (*p* < 0.05) in all three dose of AC-containing serum extracts in a dose-dependent manner. MSH2 and BRCA1 were selected as representative proteins of mismatch repair and homologous recombination, respectively, to further validate the transcriptomic prediction that AC suppresses DNA repair-associated pathways. Subsequently, we used Western blotting to detect the expression of MSH2 and BRCA1, and the results showed that AC dose-dependently suppressed the expression of MSH2 and BRCA1 (Fig. 4C–J). Collectively, these data support the findings from RNA-seq analysis and confirm that the administration of AC inhibited cell proliferation and DNA repair in CRC cells, at least in part, through the coordinated downregulation of these cell cycle regulators.

### AC enhances the anti-tumor efficacy of 5-FU by downregulating the expression of DNA repair and cell cycle related genes

**Fig. 5:**
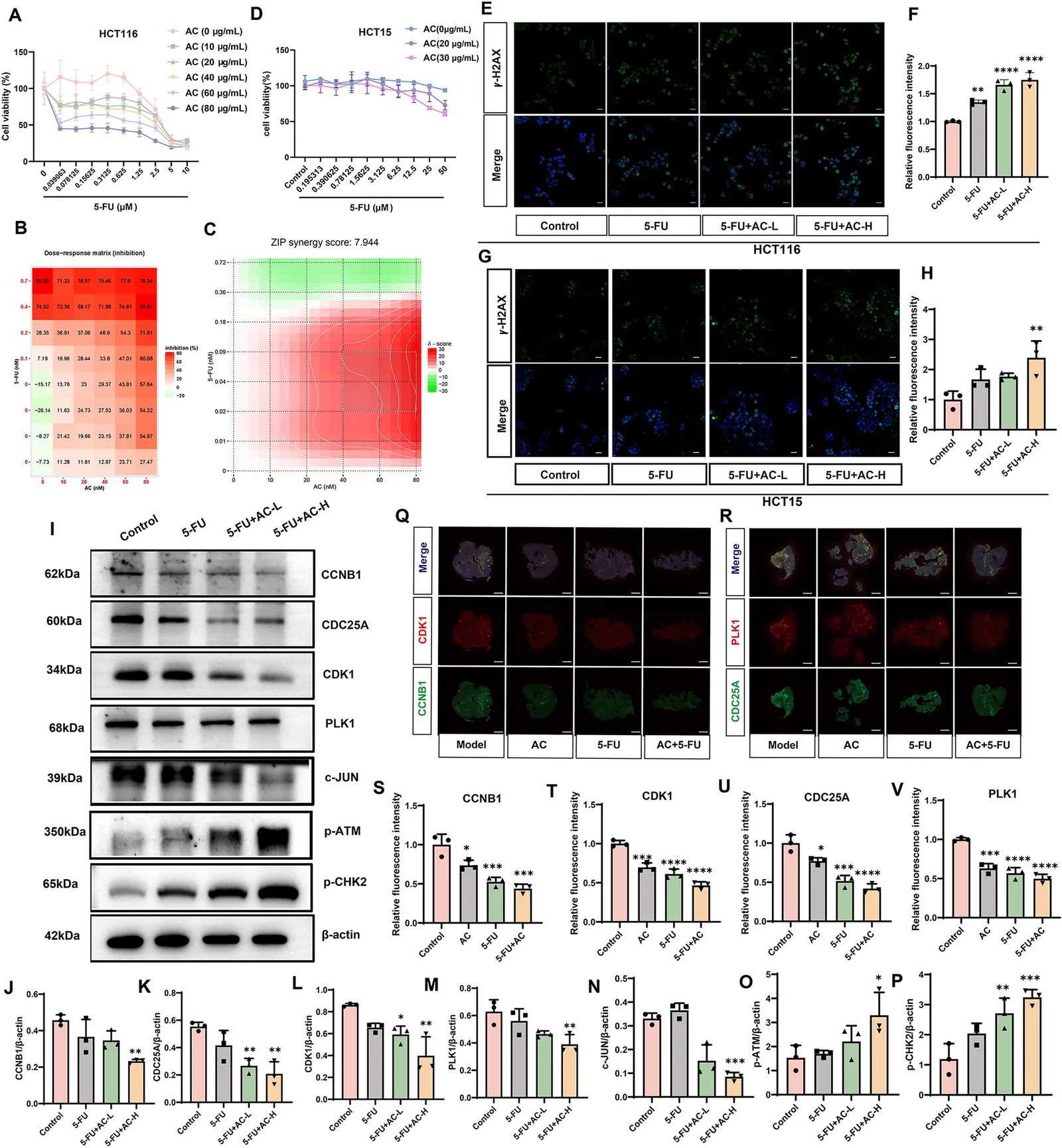
Effect of AC and 5-FU on HCT116 cells. **A**–**C** In the CCK-8 assay, AC-containing mouse serum extracts increased the inhibition of 5-FU in the HCT116 cell, and AC has a synergistic effect with 5-FU according to the synergy score calculated with SynergyFinder 3.0. **D** In the CCK-8 assay, mouse serum extract containing AC enhanced the inhibitory effect of 5-FU on HCT15 cells. **E**, **F** The combined administration of AC-containing mouse serum extracts with 5-FU significantly increased expression of γ-H2AX in the HCT116. (Scale bar: 20 μm). **G**, **H** The combined administration of AC-containing mouse serum extracts with 5-FU significantly increased expression of γ-H2AX in the HCT15. **I**–**P** Western blot analysis and quantification of CCNB1, CDC25A, CDK1, PLK1, c-JUN, p-ATM, and p-CHK2 in HCT116 cells after treatment with 5-FU alone or in combination with AC-containing serum extracts (n = 3). **Q**–**V** Immunofluorescence images and quantification showing CCNB1, CDK1, CDC25A, and PLK1 expression in xenograft tumor tissues (Scale bar: 2000 μm) (n = 3). Data are presented as the mean ± SD. Statistical significance was determined versus the model group (*p < 0.05, **p < 0.01, ***p < 0.001 ****p < 0.0001)

According to the results that AC downregulates genes governing DNA stabilization and cell proliferation, we hypothesized that AC could enhance the efficacy of chemotherapeutic agents like 5-FU, which primarily targets DNA synthesis and the cell cycle. We first evaluated the synergistic effects of serum extracts from AC-containing mice and 5-FU on HCT116 cells. The CCK-8 assay confirmed that AC significantly increased the inhibition of 5-FU on HCT116 cells. This synergistic interaction was further supported by synergy scores calculated using SynergyFinder 3.0 (Fig. 5A–C). Moreover, the combination of AC with a low dose of 5-FU markedly increased the expression of γ-H2AX, a molecular marker of DNA double-strand breaks (Fig. 5E, F). This conclusion was also validated in CT26 cells. (Supplementary Fig. 4). HCT15 cell line, harboring both p53 and KRAS mutations, was also used to investigate the synergistic effect of AC with 5-FU. We first used the CCK-8 assay to evaluate the effects of 5-FU monotherapy and the combination of AC and 5-FU on HCT15 cell viability. The results showed that HCT15 cells exhibited low sensitivity to 5-FU monotherapy, whereas cell viability was significantly reduced following combination with AC (Fig. 5D, Supplementary Fig. 5C), and DNA damage markers were significantly elevated (Fig. 5G, H). These results suggest that AC enhances the sensitivity of HCT15 cells to 5-FU.

To elucidate the mechanism underlying this synergy, we examined the expression of key cell cycle regulators following co-treatment with AC-containing mouse serum extracts and 5-FU in vitro. Western blot analysis revealed that compared with the control group or the 5-FU group, the combined therapy group-particularly the group receiving high-dose AC (30 μg/mL)-exhibited statistically significant decreases in the expression levels of c-Jun, CDK1, CDC25A, CCNB1, and PLK1 (*p* < 0.05) (Fig. 5I–N). In parallel, the levels of p-ATM and p-CHK2 were increased after combined treatment, suggesting activation of DNA damage response signaling (Fig. 5I–P). These findings indicate that AC enhances the efficacy of 5-FU by suppressing cell-cycle progression and stress-responsive regulatory proteins while promoting accumulation of DNA damage signals.

Consistent with the in vitro findings, immunofluorescence analysis of tumor tissues from the xenograft model showed that AC and 5-FU monotherapy reduced the expression of cell-cycle regulatory proteins, including CCNB1, CDK1, CDC25A, and PLK1, whereas the combination treatment produced the most pronounced inhibitory effect (Fig. 5Q–V). These in vivo results further support the notion that AC potentiates 5-FU efficacy by disrupting cell-cycle regulatory programs associated with tumor proliferation.

In conclusion, these data demonstrate that AC enhanced the therapeutic efficacy of 5-FU in CRC. This synergy is mediated, at least in part, through the downregulation of DNA repair and cell cycle regulatory genes, which enhanced DNA damage and cell cycle arrest.

### Identification of AC metabolites in vivo

**Fig. 6:**
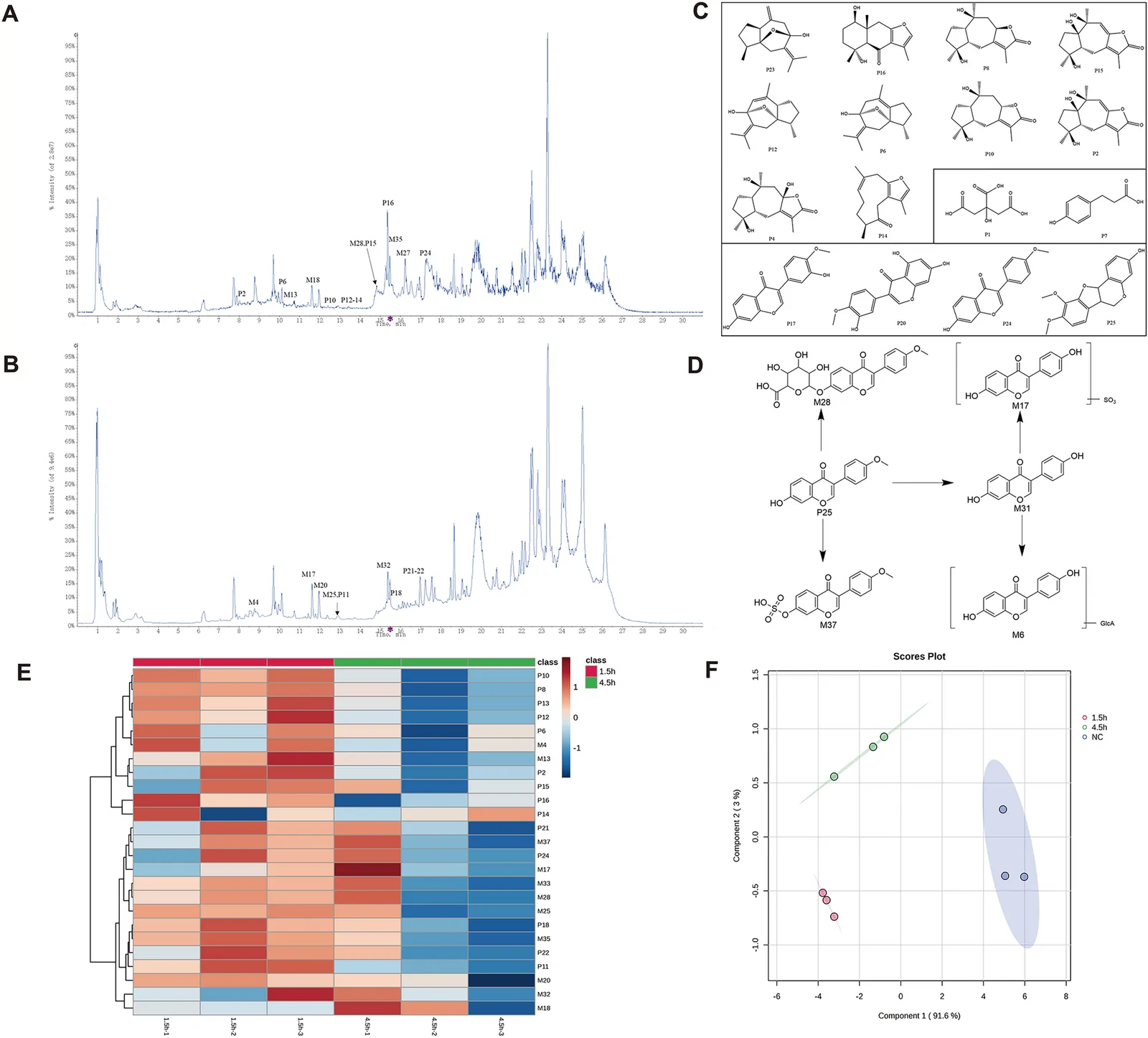
Identification of AC Metabolites in vivo. **A** The serum samples in positive ion mode. **B** The serum samples in negative ion mode. **C** The structural diagrams of representative components of prototypical components identified in rat biological samples after gavage of the aqueous extract of AC. **D** Proposed metabolic pathway of formononetin after oral administration of AC extract. **E**, **F** The PLS-DA score plot and heatmap of the metabolites identified the AC in rat serum samples after gavage of the aqueous extract of AC

To characterize the bioavailable constituents potentially responsible for the pharmacological activity of AC, we systematically profiled AC-derived components in rat serum, urine, and feces following oral administration using UPLC-QTOF-MS in both positive and negative ion modes (Fig. 6A, B, Supplementary Fig. 6A–D) [25]. Among these, 14 were detected in serum, 20 in urine, and 14 in feces. The prototype components comprise 18 sesquiterpenes (P2-P6, P8-P10, P12-P16, P18, P19, P21-P23), 3 organic acids (P1, P7, P11), and 4 flavonoids (P17, P20, P24, P25), with sesquiterpenes exhibiting the highest detection frequency. The structural diagrams of representative components are illustrated in Fig. 6C. These findings reveal a diverse array of absorbable prototype components from AC, indicating their metabolic stability and providing a basis for subsequent pharmacodynamic studies. A proposed metabolic pathway of formononetin is illustrated in Fig. 6D.

To further assess the temporal dynamics of AC-derived metabolites, multivariate statistical analysis was performed. Partial least squares-discriminant analysis (PLS-DA) of serum samples revealed a clear separation between control animals and AC-treated animals at both 1.5 h and 4.5 h post-administration (Fig. 6E), confirming that the serum metabolites originated from AC and changed dynamically between 1.5 and 4.5 h. Heatmap analysis further indicated that most serum metabolites peaked at 1.5 h (Fig. 6F), suggesting rapid absorption and early detection of AC components. Metabolite profiles between 0–6 h and 6–12 h are partially overlapped in urine (Supplementary Fig. 6E) which indicate similar metabolite composition across these intervals. Heatmap analysis showed that different metabolites varied over time without clustering within specific periods, likely reflecting distinct renal processing rates among AC constituents (Supplementary Fig. 6E). The fecal metabolites analysis showed partial overlap among the control group, 0–6 h group, and 12–24 h group with significant differences between the 0–6 h and 6–12 h groups, indicating substantial individual variation. In contrast, the 12–24 h group differed substantially from the earlier time points and showed minimal overlap with the control, suggesting that endogenous background interference was relatively limited during this interval (Supplementary Fig. 6F). Heatmap analysis further confirmed that more fecal metabolites were detected within 12–24 h, with considerable inter-individual variation, consistent with PLS-DA results. (Supplementary Fig. 6F).

These results demonstrate that multiple AC-derived constituents are rapidly absorbed into systemic circulation and remain detectable within a pharmacologically relevant time frame. The identification of serum-exposed flavonoids and sesquiterpenes provides a rational chemical basis for the observed transcriptional regulation of cell-cycle and DNA replication pathways, and supports subsequent target prediction and functional screening aimed at elucidating the molecular mediators of AC-5-FU synergistic effects.

### Identification of key compounds in AC and their synergistic effects with 5-FU

**Fig. 7:**
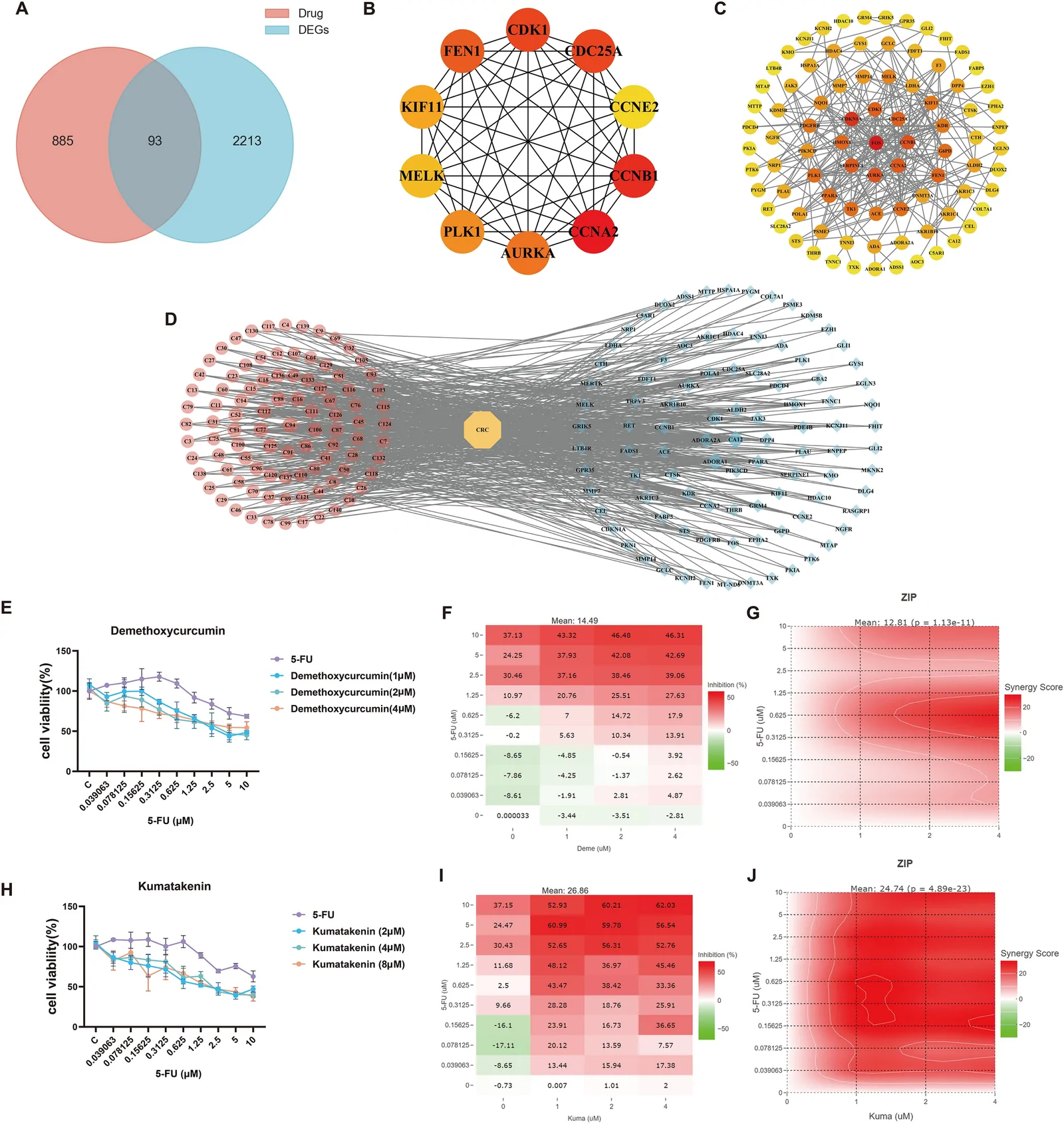
Key genes and compounds were identified based on AC and prior transcriptomic data, and the effects of compounds on key genes were validated. **A** Overlapping targets between DEGs and potential targets of candidate compounds collected from AC. **B**, **C** A PPI network diagram was generated via String network analysis, and the top ten key targets were screened using Cytohubba. **D** Network pharmacology screened compounds associated with the 93 targets, selecting the top 10 compounds with higher degree scores. **E**–**J** In CCK-8 assays, Kumatakenin and Demethoxycurcumin enhanced the inhibitory effect of 5-FU on HCT116 cells. Synergy scores calculated using SynergyFinder 3.0 indicated that both Kumatakenin and Demethoxycurcumin exhibited synergistic effects with 5-FU

A network pharmacology-based method was performed to predict the potential targets for the AC prototype compounds detected in serum. These predicted targets were then intersected with the differentially expressed genes obtained from RNA-seq in our study, yielding 93 overlapping targets (Fig. 7A) and prioritized the top ten candidate compounds based on their network degrees (Fig. 7B–D). To assess their bioactivity, these candidate compounds were functionally screened at the cellular level. The CCK-8 assay revealed that Demethoxycurcumin and Kumatakenin significantly inhibited the viability of HCT116 cells (Supplementary Fig. 5A, B). We further quantitatively evaluated their synergistic potential with 5-FU. Treatment of HCT116 cells with a range of concentrations of 5-FU (0–10 μM), in combination with either Demethoxycurcumin or Kumatakenin, demonstrated a clear synergistic enhancement of the anti-proliferative effect (Fig. 7E–J). While 5-FU alone induced only a modest reduction in cell viability, its combination with either natural compound led to a significant, dose-dependent increase in efficacy. This potent cooperative interaction was quantitatively confirmed by high synergy scores calculated using SynergyFinder 3.0.

In summary, by integrating network pharmacology with functional validation, we identified Demethoxycurcumin and Kumatakenin as two active constituents within the complex AC mixture that mediate its synergistic enhancement of 5-FU efficacy in CRC cells.

### Demethoxycurcumin and Kumatakenin simulate the cell cycle-arresting effects of AC

**Fig. 8:**
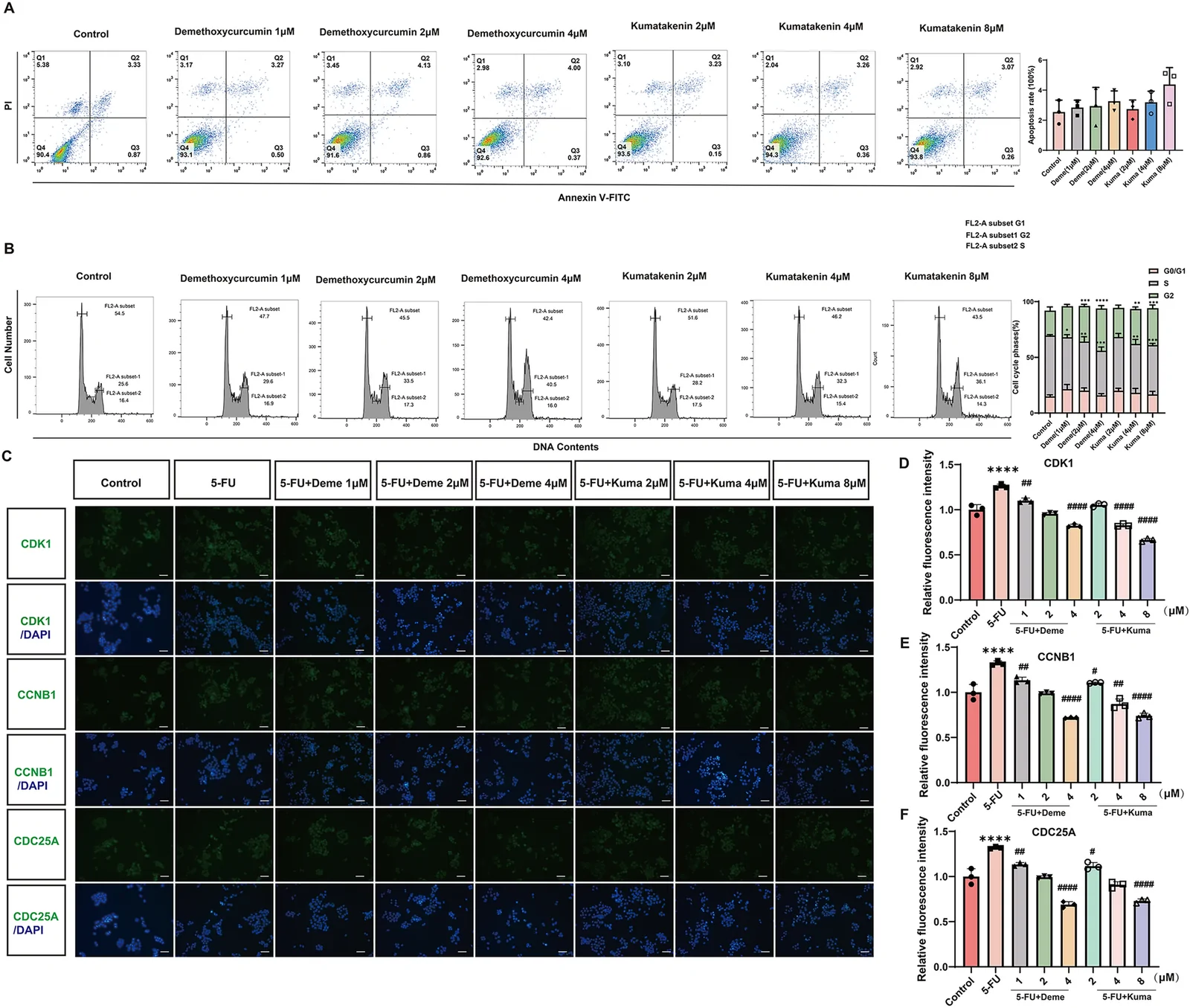
Validation of the effects of different concentrations of Demethoxycurcumin and Kumatakenin on apoptosis, cell cycle progression, and related proteins in HCT116 cells. **A**, **B** Flow cytometry analysis of the effects of different doses of Kumatakenin and Demethoxycurcumin on apoptosis and cell cycle progression in HCT116 cells. **C**–**F** Immunofluorescence images show the expression levels of CDK1, CCNB1, and CDC25A in HCT116 cells treated with 5-FU (1 μM) alone and with 5-FU (1 μM) combined with different concentrations of Kumatakenin and Demethoxycurcumin (n = 3). (Scale bar: 200 μm). Data are presented as the mean ± SD (compared with the control group, ****p < 0.0001; compared with the 5-FU group, ###p < 0.001, ####p < 0.0001)

To determine whether the identified core compounds, Demethoxycurcumin and Kumatakenin, directly simulate the biological effects of the full AC extract, we investigated their influence on apoptosis and the cell cycle in HCT116 cells. The results showed that neither Demethoxycurcumin nor Kumatakenin significantly induced apoptosis in HCT116 cells (Fig. 8A). In contrast, both compounds potently interfered with cell cycle progression. Similar to the AC extract, treatment with Demethoxycurcumin or Kumatakenin induced a dose-dependent G2/M-phase arrest (Fig. 8B). We further evaluated the expression of key cell cycle regulatory proteins. Immunofluorescence analysis demonstrated that the combination of either compound with 5-FU effectively suppressed the expression of CDK1, CCNB1, and CDC25A in HCT116 cells (Fig. 8C–F). Additionally, we investigated the effects of Demethoxycurcumin and Kumatakenin on the cell cycle and apoptosis in CT26 cells. (Supplementary Fig. 7A-F) The results were consistent with those observed in HCT116 cells.

Collectively, these data demonstrate that Demethoxycurcumin and Kumatakenin are functionally active components within AC. They mirror the extract's ability to synergize with 5-FU by modulating the cell cycle, thereby contributing to the inhibition of colorectal cancer progression.

### Molecular docking and molecular dynamics simulations evaluate the interactions of Demethoxycurcumin and Kumatakenin with CCNB1, CDK1, and CDC25A

**Fig. 9:**
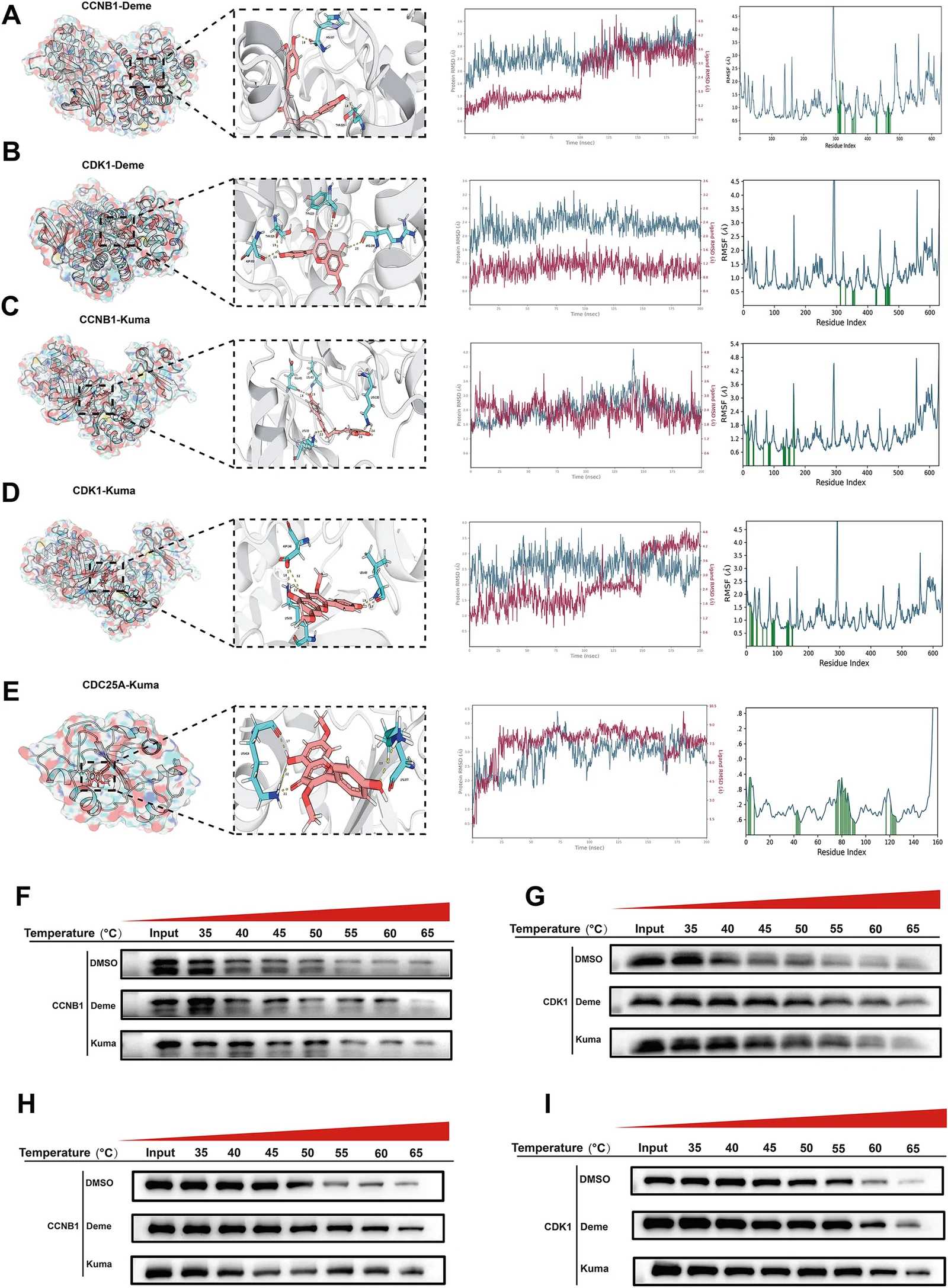
The binding patterns of Kumatakenin, Demethoxycurcumin, and CCNB1, CDK1, CDC25A. **A**–**E** The stability of ligand-target complexes between these compounds with CCNB1, CDK1, and CDC25A was evaluated by protein–ligand root mean square deviation (RMSD) during molecular dynamics simulations. **F**, **G** CETSA validated the targeted binding of Demethoxycurcumin and Kumatakenin to CCNB1 and CDK1 in HCT116 cells. **H**, **I** CETSA validated the targeted binding of Demethoxycurcumin and Kumatakenin to purified recombinant CCNB1 and CDK1

To evaluate the potential interactions between Kumatakenin or Demethoxycurcumin and the candidate targets, CCNB1, CDK1, CDC25A, molecular docking was performed using Maestro. The docking scores for Kumatakenin-CCNB1, Kumatakenin-CDK1, Kumatakenin-CDC25A, Demethoxycurcumin-CCNB1, Demethoxycurcumin-CDK1, and Demethoxycurcumin-CDC25A were −7.325, −7.165, −5.124, −7.145, −6.264, and −4.867 kcal/mol (Supplementary Table 1). Subsequently, we selected compounds with docking scores below −5 kcal/mol for molecular dynamics simulations. Based on docking scores and MD simulations, Kumatakenin formed tightly stable interactions with CCNB1, CDK1, and CDC25A. Demethoxycurcumin also exhibited stable binding with CCNB1. However, its binding to CDK1 involved significant conformational changes in the protein–ligand complex, resulting in unstable interactions (Fig. 9A–E). To verify the computer simulation analysis, we adopted the cellular thermal shift assay (CETSA) (Fig. 9F, G). In DMSO-treated lysates, CCNB1 and CDK1 levels decreased with increasing temperature, whereas treatment with Kumatakenin or Demethoxycurcumin increased the thermal stability with higher levels of soluble CCNB1 and CDK1 at elevated temperatures. We further performed purified recombinant protein-based CETSA using CCNB1 and CDK1. Under cell-free conditions, Demethoxycurcumin and Kumatakenin increased the thermal stability of the recombinant proteins, supporting direct target engagement and reducing the possibility that the stabilization observed in cell lysates was solely mediated by indirect cellular factors (Fig. 9H, I).

## Discussion

Colorectal cancer (CRC) remains a major global health burden and one of the leading causes of cancer-related mortality worldwide [26]. Although surgery, chemotherapy, targeted therapy, and immunotherapy have improved patient outcomes, resistance to fluoropyrimidine-based chemotherapy, particularly 5-FU, continues to limit durable therapeutic benefit [27, 28]. Increasing evidence indicates that DNA repair capacity, cell-cycle checkpoint regulation, and adaptive survival signaling strongly influence CRC response to chemotherapy [29]. In the present study, we demonstrated that AC potentiated the antitumor efficacy of 5-FU in CRC models. Mechanistically, AC appeared to weaken DNA repair-cell-cycle coordination and stress-survival signaling, thereby increasing the vulnerability of CRC cells to 5-FU-induced replication stress and DNA damage.

Transcriptomic profiling revealed that AC broadly suppressed gene networks associated with DNA replication, mismatch repair, and cell-cycle progression. These findings are consistent with the concept that rapidly proliferating cancer cells depend on coordinated DNA synthesis, DNA repair, and checkpoint control to maintain genome stability under therapeutic stress [29–31]. The coordinated downregulation of PLK1, CDK1, CCNB1, CDC25A, JUN, MSH2 and BRCA1 suggests that AC does not act through a single molecular target but perturbs a regulatory network required for genome maintenance and proliferative adaptation. PLK1, CDK1, CCNB1 and CDC25A form a core mitotic regulatory module that controls G2/M transition, checkpoint recovery, and mitotic progression [31–33]. MSH2 and BRCA1 were selected as representative proteins of mismatch repair and homologous recombination, respectively, to support the transcriptomic prediction that AC suppresses DNA repair-associated pathways. c-JUN, as a component of the activator protein-1 transcription factor complex, functions as a stress-responsive transcriptional regulator, and KRAS-related transcriptional programs involving JUN have been implicated in CRC biology [34]. Therefore, simultaneous suppression of these molecules provides a mechanistic explanation for the observed cell-cycle arrest and enhanced DNA damage after the combination of AC and 5-FU.

The relevance of this network-level regulation is particularly important in the context of 5-FU treatment. 5-FU exerts its antitumor activity partly by inhibiting thymidylate synthesis and disrupting DNA replication, while its metabolites can also be incorporated into RNA and DNA [3]. Recent evidence further indicates that RNA damage responses, including effects on ribosome biogenesis, contribute to 5-FU-mediated lethality in CRC [4]. Tumor cells may survive these stresses by activating DNA repair pathways, checkpoint adaptation, and pro-survival signaling. In our study, AC enhanced the inhibitory effect of 5-FU and increased γ-H2AX accumulation, suggesting that AC may aggravate 5-FU-induced DNA damage or impair cellular adaptation to such damage. The validation of MSH2 and BRCA1 further supports the involvement of DNA repair-associated pathways. In addition to γ-H2AX accumulation, the increased levels of p-ATM and p-CHK2 after combined treatment further indicate activation of DNA damage response signaling. Importantly, these markers should be interpreted as evidence of increased DNA damage burden and checkpoint activation.

The present study also clarifies the relationship between cell-cycle arrest and apoptosis after AC treatment. Previous studies have shown that 5-FU can induce apoptosis in CRC cells through caspase-dependent pathways and mitochondrial dysfunction [35, 36]. However, in the present study, AC and its active compounds primarily induced G2/M-phase arrest at early time points, whereas apoptosis was limited initially and became more evident only after prolonged treatment. In addition, G2/M-phase arrest was at least partially reversible after drug withdrawal. These findings suggest that AC does not immediately act as a direct cytotoxic agent but instead imposes a transient cell-cycle blockade and weakens cellular stress adaptation. Under this condition, 5-FU-induced damage may accumulate more efficiently, and apoptosis may occur as a delayed secondary consequence rather than the primary early mechanism of AC action.

The suppression of cell-cycle regulators observed in this study is closely related to chemotherapy sensitization. CDK1 has been reported to contribute to 5-FU resistance in CRC, and inhibition of CDK1 can reverse 5-FU resistance [37]. PLK1 is associated with chromosomal instability, checkpoint override, and tumor progression [33], and PLK1 inhibition has been shown to reverse 5-FU resistance in CRC cells [7]. Clinically, the PLK1 inhibitor on vansertib has been explored in combination with FOLFIRI and bevacizumab in the second-line treatment of KRAS-mutant metastatic CRC, supporting the translational relevance of PLK1 as a chemotherapy-sensitizing target [38]. CDC25A is another important cell-cycle regulator linked to chemoresistance, and B7-H3-mediated CRC chemoresistance has been reported to involve CDC25A regulation [8]. In addition, inhibition of ATR kinase has been shown to enhance 5-FU sensitivity, further supporting the importance of checkpoint adaptation in 5-FU response [39]. Together, these findings support the interpretation that AC enhances 5-FU efficacy by weakening cell-cycle checkpoint adaptation and DNA damage tolerance.

Functional validation further supported the involvement of c-JUN and AKT signaling in AC-mediated 5-FU sensitization. c-JUN overexpression partially attenuated the sensitizing effect of AC (Supplementary Fig. 8), suggesting that c-JUN downregulation contributes to the enhanced 5-FU response. In parallel, AC reduced p-AKT levels, and pharmacological AKT inhibition partially mimicked the sensitizing effect of AC (Supplementary Fig. 9). These observations are consistent with the broader role of survival signaling in 5-FU resistance [28, 40]. However, these data should be interpreted as supportive rather than definitive proof of a linear causal pathway. Additional rescue experiments, such as constitutively active AKT expression, AKT reactivation, c-JUN knockdown, and pathway epistasis analyses, will be required to determine the precise net relationship among c-JUN, AKT, DNA repair, and cell-cycle regulatory networks.

In addition to HCT116 and CT26 cells, validation in HCT15 cells provided further evidence that AC can enhance 5-FU sensitivity in a genetically distinct CRC background. This is relevant because genetic heterogeneity, including alterations in KRAS and TP53-related signaling, strongly influences chemotherapy response [41, 42]. Nevertheless, CRC is highly heterogeneous, and further validation across a broader panel of CRC cell lines, patient-derived organoids, and patient-derived xenograft models will be necessary to determine whether the AC-5-FU interaction is broadly applicable or more effective in specific molecular subtypes.

Through metabolite profiling, network pharmacology, and functional screening, Demethoxycurcumin and Kumatakenin were identified as bioavailable active constituents that contribute to the pharmacological activity of AC. Both compounds inhibited CRC cell proliferation, induced G2/M-phase arrest, and enhanced the antiproliferative effect of 5-FU. Previous evidence has also shown that curcumin-related compounds can cooperate with 5-FU in colon cancer cells [14, 43], supporting the plausibility that bioavailable constituents from *Curcumae Rhizoma* may contribute to 5-FU sensitization. Molecular docking and molecular dynamics simulations suggested stable interactions with selected cell-cycle targets. CETSA in cell lysates supported target engagement, and additional assays using purified recombinant CCNB1 and CDK1 proteins further reduced the possibility that the thermal stabilization was solely mediated by indirect cellular factors. Nevertheless, because CETSA does not provide quantitative binding kinetics or binding-site information, future studies using surface plasmon resonance, isothermal titration calorimetry, competition assays, or target-mutant validation are still needed to further define binding affinity and specificity.

It should also be emphasized that Demethoxycurcumin and Kumatakenin only partially recapitulate the activity of the complete AC extract. AC is a complex multi-component herbal formula, and additional absorbed constituents may contribute to its biological effects through complementary or synergistic mechanisms. Several compounds derived from *Astragali Radix* or *Curcumae Rhizoma*, including curcumin-related constituents, curcumol, and formononetin, have been reported to exert anticancer effects through multiple molecular pathways [13, 14, 44]. Therefore, although Demethoxycurcumin and Kumatakenin are important bioavailable contributors, they should not be regarded as complete substitutes for AC. Direct comparison of Demethoxycurcumin plus Kumatakenin with the full AC extract will be important for determining whether other constituents contribute to the overall chemosensitizing activity. In addition, the relationship between the in vitro concentrations of these compounds and their serum exposure levels requires further pharmacokinetic clarification before translational conclusions can be made.

From a translational perspective, these findings suggest that AC or its active constituents may be developed as adjunctive sensitizing strategies for 5-FU-based chemotherapy. 5-FU is commonly used as a backbone component of standard regimens such as FOLFOX and FOLFIRI, and herbal medicine combined with FOLFOX has previously been evaluated in advanced CRC [11]. Therefore, future preclinical studies should evaluate whether AC can enhance the efficacy of clinically relevant regimens such as FOLFOX or FOLFIRI without increasing systemic toxicity. In the present study, serum biochemical analyses suggested that AC did not exacerbate 5-FU-associated hepatic or renal biochemical toxicity under the tested conditions. However, more comprehensive safety evaluation, including long-term body weight monitoring, liver and kidney histology, hematological toxicity, intestinal mucosal injury, and pharmacokinetic interaction with 5-FU, will be required before clinical translation. Future studies may test AC in combination with FOLFOX in orthotopic CRC models, metastatic models, patient-derived organoids, and patient-derived xenografts, with particular attention to dose optimization, treatment sequence, and the possibility of reducing fluoropyrimidine or oxaliplatin dose intensity.

Several limitations should be acknowledged. First, although AC downregulated representative proteins involved in cell-cycle regulation and DNA repair, direct functional assays of DNA repair capacity were not performed. Second, c-JUN overexpression and AKT inhibition supported the involvement of stress-responsive transcriptional and survival signaling, but further gain- and loss-of-function rescue experiments are needed to define causal relationships. Third, Demethoxycurcumin and Kumatakenin only partially represent the chemical complexity of AC, and the contribution of other bioavailable constituents remains to be clarified. Fourth, although cell lysate-based and purified protein-based CETSA supported target engagement, quantitative biophysical assays and binding-site validation were not performed.

In conclusion, this study demonstrates that AC enhances the antitumor efficacy of 5-FU in CRC by weakening DNA repair-cell-cycle coordination and stress-survival signaling. By suppressing key regulators such as PLK1, CDK1, CCNB1, CDC25A, JUN, MSH2, BRCA1 and p-AKT, AC increases 5-FU-induced DNA damage and promotes cell-cycle arrest. Demethoxycurcumin and Kumatakenin were identified as important bioavailable constituents that partially recapitulate the chemosensitizing effect of AC. These findings provide a mechanistic basis for the traditional use of AC as a chemoadjuvant and support its further development as a multi-component sensitizing strategy for CRC chemotherapy.

## Conclusions

In summary, this study demonstrates that the *Astragali Radix-Curcumae Rhizoma* herb pair potentiates the antitumor efficacy of 5-FU in colorectal cancer. Mechanistically, AC weakens DNA repair-cell-cycle coordination and stress-survival signaling by downregulating key regulators associated with cell-cycle progression, DNA repair, and chemoresistance, including JUN, CCNB1, CDC25A, PLK1, CDK1, MSH2, BRCA1, and p-AKT. This network regulation increases 5-FU-induced DNA damage and promotes cell-cycle arrest, thereby enhancing CRC sensitivity to 5-FU. Demethoxycurcumin and Kumatakenin were identified as bioavailable active constituents that partially recapitulate the sensitizing effect of AC. These findings provide a mechanistic basis for the use of AC as an adjunctive strategy to improve 5-FU-based chemotherapy in CRC.

## Supplementary Information

Supplementary material 1: Figure 1. The typical overlaid extracted ion chromatograms of 44 identified constituents from the aqueous extract of AC herbal pairs in positive (A) and negative (B) ion mode. Red label means the identification was confirmed by comparison with reference standards.

Supplementary material 2: Figure 2. Effects of the Combination of AC and 5-FU on Biochemical Markers of Liver and Kidney Function in Mouse Serum. (A-D) Effects of the Combination of AC and 5-FU on Changes in ALT, AST, BUN, and CREA Levels. (n = 3).

Supplementary material 3: Figure 3. The effects of rat serum extracts containing AC at various concentrations, Kumatakenin, and Demethoxycurcumin on the cell cycle, and the effects of AC-containing serum extracts at various concentrations on apoptosis (A) Effects of rat serum extracts treated with different concentrations of AC on the cell cycle 24 h after treatment and 24 h after withdrawal. (B) The effects of 24-hour treatment with different concentrations of Kumatakenin and Demethoxycurcumin, followed by a 24-hour washout period, on the cell cycle. (C) The effect of 72-hour treatment with different concentrations of AC rat serum extract on cell apoptosis. (D) Statistical graph showing the effects of AC rat serum extract at different concentrations on cell apoptosis after treatment for varying durations. Data are presented as the mean ± SD. (Compared with the control group, **p < 0.01, ***p < 0.001, ****p < 0.0001).

Supplementary material 4: Figure 4. The combined administration of AC with 5-FU significantly increased expression of γ-H2AX. (n = 3). (Scale bar: 20 μm). Data are presented as the mean ± SD. (Compared with the control group, *p < 0.05, **p < 0.01, ***p < 0.001, ****p < 0.0001).

Supplementary material 5: Figure 5. Using CCK-8 assay, we evaluated the effects of selected compounds with higher Degree scores from Figure 7 on the viability of HCT116 cells. (A)CCK-8 results for HCT116 cells treated with 5-FU alone versus Isomucronulatol, Demethoxycurcumin, Naringenin, Methylnissolin, Apigenin, Formononetin, and Kumatakenin. (B)CCK-8 assay results for HCT116 cells treated with 10 μM 5-FU in combination with various concentrations of Isomucronulatol, Demethoxycurcumin, Naringenin, Methylnissolin, Apigenin, Formononetin, and Kumatakenin. (n = 3). Data are presented as the mean ± SD. (Compared with the control group, *p < 0.05, **p < 0.01, ***p < 0.001, ****p < 0.0001).

Supplementary material 6: Figure 6. Identification of AC Metabolites in *vivo.* (A) The urine samples in positive ion mode. (B) The urine samples in negative ion mode. (C) The feces samples in positive ion mode. (D) The feces samples in negative ion mode. (E) The PLS-DA score plot and heatmap of the metabolites identified the AC in rat urine samples after gavage of the aqueous extract of AC. (F) The PLS-DA score plot and heatmap of the metabolites identified the AC in rat feces samples after gavage of the aqueous extract of AC.

Supplementary material 7: Figure 7. Validation of the effects of different concentrations of Demethoxycurcumin and Kumatakenin on apoptosis, cell cycle progression, and related proteins in CT26 cells. (A-B) Flow cytometry analysis of the effects of different doses of Kumatakenin and Demethoxycurcumin on apoptosis and cell cycle progression in CT26 cells. (C-F) Immunofluorescence images show the expression levels of CDK1, CCNB1, and CDC25A in CT26 cells treated with 5-FU alone and with 5-FU combined with different concentrations of Kumatakenin and Demethoxycurcumin. (n = 3). (Scale bar: 200 μm). Data are presented as the mean ± SD. (Compared with the control group, *p < 0.05, **p < 0.01, ***p < 0.001, ****p < 0.0001; compared with the 5-FU group, ^#^*p *< 0.05, ^##^*p *< 0.01, ^###^ *p*< 0.001, ^####^ *p*< 0.0001).

Supplementary material 8: Figure 8. Validation of the findings regarding c-JUN's involvement in AC-mediated 5-FU sensitization. (A) The HCT116 cell line, which stably overexpresses c-JUN, was identified and selected through immunofluorescence verification. (B) Western blot analysis of c-JUN protein levels in the overexpression group. (C) Assessment of the effect of AC combined with 5-FU treatment on the viability of c-JUN-overexpressing cells using the CCK-8 assay.

Supplementary material 9: Figure 9. To determine whether AKT inhibitors can mimic the sensitizing effect of AC on 5-FU. (A) CCK-8 assay to evaluate the effects of AKT inhibitors and 5-FU, both as monotherapies and in combination, on cell viability. (B) The combined administration of AKT inhibitors with 5-FU significantly increased expression of γ-H2AX.

Supplementary material 10: Table 1. Molecular docking scores of Kumatakenin and Demethoxycurcumin withCCNB1, CDK1, and CDC25A.

## Data Availability

No datasets were generated or analysed during the current study.

## Abbreviations

5-FU: 5-Fluorouracil
AC: *Astragali Radix*–*Curcumae Rhizoma*
AP-1: Activator protein 1
Bax: Bcl-2-associated X-protein
Bcl-2: B-cell lymphoma protein 2
BPs: Biological processes
CCNB1: Cyclin B1
CCs: Cell components
CDC25A: Cell division cycle 25A
CDK1: Cyclin-dependent kinase 1
CRC: Colorectal cancer
CMC-Na: Carboxymethylcellulose sodium
DEGs: Differentially expressed genes
Deme: Demethoxycurcumin
EZ: *Curcumae Rhizoma*
GO: Gene ontology
HQ: *Astragali Radix*
IF: Immunofluorescence
JUN: Jun proto-oncogene
KEGG: Kyoto Encyclopedia of Genes and Genomes
Kuma: Kumatakenin
MFs: Molecular functions
NPT: Number of particles, pressure and temperature
OD: Optical density
OPLS4: Optimized potential for liquid simulations 4
p-AKT: Phosphorylated protein kinase B
PLK1: Polo like kinase 1
PLS-DA: Partial least squares-discriminant analysis
RIN: RNA integrity number
RNA-seq: RNA sequencing
SPC: Simple point charge
TCM: Traditional Chinese medicine
GEO: Gene Expression Omnibus

## References

[1] Bray F, Laversanne M, Sung H, Ferlay J, Siegel RL, Soerjomataram I, et al. Global cancer statistics 2022: GLOBOCAN estimates of incidence and mortality worldwide for 36 cancers in 185 countries. CA Cancer J Clin. 2022;74(3):229–63. 10.3322/caac.21834. 38572751

[2] Morris VK, Kennedy EB, Baxter NN, Benson AB 3rd, Cercek A, Cho M, et al. Treatment of metastatic colorectal cancer: ASCO guideline. J Clin Oncol. 2023;41(3):678–700. 10.1200/jco.22.01690. PMC10506310 36252154

[3] Vodenkova S, Buchler T, Cervena K, Veskrnova V, Vodicka P, Vymetalkova V. 5-fluorouracil and other fluoropyrimidines in colorectal cancer: Past, present and future. Pharmacol Ther. 2020;206:107447. 10.1016/j.pharmthera.2019.107447. 31756363

[4] Chen JK, Merrick KA, Kong YW, Izrael-Tomasevic A, Eng G, Handly ED, et al. An RNA damage response network mediates the lethality of 5-FU in colorectal cancer. Cell Rep Med. 2024;5(10):101778. 10.1016/j.xcrm.2024.101778. PMC11514606 39378883

[5] Simoneau A, Zou L. An extending ATR-CHK1 circuitry: the replication stress response and beyond. Curr Opin Genet Dev. 2021;71:92–8. 10.1016/j.gde.2021.07.003. 34329853

[6] Yao J, Huang A, Zheng X, Liu T, Lin Z, Zhang S, et al. 53BP1 loss induces chemoresistance of colorectal cancer cells to 5-fluorouracil by inhibiting the ATM-CHK2-P53 pathway. J Cancer Res Clin Oncol. 2017;143(3):419–31. 10.1007/s00432-016-2302-5. PMC11819077 27838786

[7] Poyil PK, Siraj AK, Padmaja D, Parvathareddy SK, Alobaisi K, Thangavel S, et al. Polo-like kinase 1 predicts lymph node metastasis in Middle Eastern colorectal cancer patients; its inhibition reverses 5-Fu resistance in colorectal cancer cells. Cells. 2024. 10.3390/cells13201700. PMC11506015 39451218

[8] Ma Y, Wang R, Lu H, Li X, Zhang G, Fu F, et al. B7-H3 promotes the cell cycle-mediated chemoresistance of colorectal cancer cells by regulating CDC25A. J Cancer. 2020;11(8):2158–70. 10.7150/jca.37255. PMC7052923 32127943

[9] Wang D, Wang T, An Y, Jin L, Wang J, Wu G, et al. Nephroblastoma overexpressed protein (NOV) enhances 5-Fu-mediated inhibitory effect of colorectal cancer cell proliferation via JNK/AP-1/caspase-8/caspase-3 pathway. Discov Oncol. 2021;12(1):10. 10.1007/s12672-021-00403-y. PMC8777523 35201461

[10] Ramesh P, Medema JP. BCL-2 family deregulation in colorectal cancer: potential for BH3 mimetics in therapy. Apoptosis. 2020;25(5–6):305–20. 10.1007/s10495-020-01601-9. PMC7244464 32335811

[11] Chen MH, May BH, Zhou IW, Xue CCL, Zhang AL. FOLFOX 4 combined with herbal medicine for advanced colorectal cancer: a systematic review. Phytother Res. 2014;28(7):976–91. 10.1002/ptr.5092. 24343974

[12] Chen P, Li XJ, Zhang RN, Liu SP, Xiang Y, Zhang MM, et al. Combinative treatment of β-elemene and cetuximab is sensitive to KRAS mutant colorectal cancer cells by inducing ferroptosis and inhibiting epithelial-mesenchymal transformation. Theranostics. 2020;10(11):5107–19. 10.7150/thno.44705. PMC7163451 32308771

[13] Hashem S, Nisar S, Sageena G, Macha MA, Yadav SK, Krishnankutty R, et al. Therapeutic effects of curcumol in several diseases; an overview. Nutr Cancer. 2021;73(2):181–95. 10.1080/01635581.2020.1749676. 32285707

[14] Johnson JJ, Mukhtar H. Curcumin for chemoprevention of colon cancer. Cancer Lett. 2007;255(2):170–81. 10.1016/j.canlet.2007.03.005. 17448598

[15] Ong SKL, Shanmugam MK, Fan L, Fraser SE, Arfuso F, Ahn KS, et al. Focus on formononetin: anticancer potential and molecular targets. Cancers. 2019. 10.3390/cancers11050611. PMC6562434 31052435

[16] Cheng XD, Lu Y. A review of capecitabine-based adjuvant therapy for gastric cancer in the Chinese population. Future Oncol. 2018;14(8):771–9. 10.2217/fon-2017-0558. 29252007

[17] Gu J, Sun R, Tang D, Liu F, Chang X, Wang Q. *Astragalus mongholicus* Bunge-*Curcuma aromatica* Salisb. suppresses growth and metastasis of colorectal cancer cells by inhibiting M2 macrophage polarization via a Sp1/ZFAS1/miR-153-3p/CCR5 regulatory axis. Cell Biol Toxicol. 2022;38(4):679–97. 10.1007/s10565-021-09679-w. 35072892

[18] Bian Y, Wang G, Zhou J, Yin G, Liu T, Liang L, et al. *Astragalus membranaceus* (Huangqi) and *Rhizoma curcumae* (Ezhu) decoction suppresses colorectal cancer via downregulation of Wnt5/β-catenin signal. Chin Med. 2022;17(1):11. 10.1186/s13020-021-00564-6. PMC8740427 34991661

[19] Yang Q, Sun Z, Zhu YM, Xiang DY, Zhang QY, Wang F, et al. Astragali Radix-Curcumae Rhizoma combination inhibits proliferation, migration, and invasion of colon cancer HT-29 cells by regulating EMT. Zhongguo Zhong Yao Za Zhi. 2023;48(3):736–43. 10.19540/j.cnki.cjcmm.20221102.701. 36872237

[20] Sun R, Sun C, Yue Z, Yin G, Zhou L, Zhang S, et al. Astragali Radix-Curcumae Rhizoma herb pair reduces the stemness of colorectal cancer cells through HIF-2α/β-catenin pathway. Phytomedicine. 2024;132:155824. 10.1016/j.phymed.2024.155824. 38941816

[21] Peng W, Zhang H, Tan S, Li Y, Zhou Y, Wang L, et al. Synergistic antitumor effect of 5-fluorouracil with the novel LSD1 inhibitor ZY0511 in colorectal cancer. Ther Adv Med Oncol. 2020;12:1758835920937428. 10.1177/1758835920937428. PMC7378962 32754230

[22] Wang Y, Yuan S, Li L, Yang D, Xu C, Wang S, et al. Novel proapoptotic agent SM-1 enhances the inhibitory effect of 5-fluorouracil on colorectal cancer cells in vitro and in vivo. Oncol Lett. 2017;13(6):4762–8. 10.3892/ol.2017.6043. PMC5452999 28599477

[23] Sun RL, Tang DC, Gu JF. Study on intervention effect of Astragali Radix-Curcumae Rhizoma on growth and metastasis of colon cancer in orthotopic transplantation mice model of colon cancer. Zhongguo Zhong Yao Za Zhi. 2021;46(9):2267–75. 10.19540/j.cnki.cjcmm.20201207.402. 34047130

[24] Liu Q, Yin X, Languino LR, Altieri DC. Evaluation of drug combination effect using a Bliss independence dose-response surface model. Stat Biopharm Res. 2018;10(2):112–22. 10.1080/19466315.2018.1437071. PMC6415926 30881603

[25] He QR, Chen YC, Lu MX, Sun RQ, Wang SS, Qiu X, et al. Rapid and systematic characterization of chemical constituents in *Astragali radix-Curcumae rhizoma* herb pair using an UPLC-QTOF-MS-based strategy. Biomed Chromatogr. 2025;39(7):e70097. 10.1002/bmc.70097. 40415205

[26] Matsuda T, Fujimoto A, Igarashi Y. Colorectal cancer: epidemiology, risk factors, and public health strategies. Digestion. 2025;106(2):91–9. 10.1159/000543921. 39938491

[27] Abedizadeh R, Majidi F, Khorasani HR, Abedi H, Sabour D. Colorectal cancer: a comprehensive review of carcinogenesis, diagnosis, and novel strategies for classified treatments. Cancer Metastasis Rev. 2024;43(2):729–53. 10.1007/s10555-023-10158-3. 38112903

[28] Gmeiner WH, Okechukwu CC. Review of 5-FU resistance mechanisms in colorectal cancer: clinical significance of attenuated on-target effects. Cancer Drug Resist. 2023;6(2):257–72. 10.20517/cdr.2022.136. PMC10344727 37457133

[29] Vodicka P, Vodenkova S, Buchler T, Vodickova L. DNA repair capacity and response to treatment of colon cancer. Pharmacogenomics. 2019;20(17):1225–33. 10.2217/pgs-2019-0070. 31691643

[30] Santivasi WL, Xia F. Ionizing radiation-induced DNA damage, response, and repair. Antioxid Redox Signal. 2014;21(2):251–9. 10.1089/ars.2013.5668. 24180216

[31] Pellarin I, Dall’Acqua A, Favero A, Segatto I, Rossi V, Crestan N, et al. Cyclin-dependent protein kinases and cell cycle regulation in biology and disease. Signal Transduct Target Ther. 2025. 10.1038/s41392-024-02080-z. PMC11734941 39800748

[32] Timofeev O, Cizmecioglu O, Settele F, Kempf T, Hoffmann I. Cdc25 phosphatases are required for timely assembly of CDK1-cyclin B at the G2/M transition. J Biol Chem. 2010;285(22):16978–90. 10.1074/jbc.M109.096552. PMC2878026 20360007

[33] Gheghiani L, Wang L, Zhang YW, Moore XTR, Zhang JL, Smith SC, et al. PLK1 induces chromosomal instability and overrides cell-cycle checkpoints to drive tumorigenesis. Cancer Res. 2021;81(5):1293–307. 10.1158/0008-5472.Can-20-1377. PMC8026515 33376114

[34] Serra RW, Fang MG, Park SM, Hutchinson L, Green MR. A KRAS-directed transcriptional silencing pathway that mediates the CpG island methylator phenotype. Elife. 2014. 10.7554/eLife.02313. PMC3949416 24623306

[35] Adachi Y, Taketani S, Oyaizu H, Ikebukuro K, Tokunaga R, Ikehara S. Apoptosis of colorectal adenocarcinoma induced by 5-FU and/or IFN-γ through caspase 3 and caspase 8. Int J Oncol. 1999;15(6):1191–6. 10.3892/ijo.15.6.1191 10568827

[36] Grivicich I, Regner A, da Rocha AB, Grass LB, Alves PAG, Kayser GB, et al. Irinotecan/5-fluorouracil combination induces alterations in mitochondrial membrane potential and caspases on colon cancer cell lines. Oncol Res. 2005;15(7–8):385–92. 10.3727/096504005776449680. 16491956

[37] Zhu YP, Li K, Zhang JL, Wang L, Sheng LL, Yan L. Inhibition of CDK1 reverses the resistance of 5-Fu in colorectal cancer. Cancer Manag Res. 2020;12:11271–83. 10.2147/cmar.S255895. PMC7649235 33177877

[38] Ahn DH, Ridinger M, Cannon TL, Mendelsohn L, Starr JS, Hubbard JM, et al. Onvansertib in combination with chemotherapy and bevacizumab in second-line treatment of KRAS-mutant metastatic colorectal cancer: a single-arm, phase II trial. J Clin Oncol. 2025. 10.1200/jco-24-01266. PMC11856007 39475591

[39] Ito SS, Nakagawa Y, Matsubayashi M, Sakaguchi YM, Kobashigawa S, Matsui TK, et al. Inhibition of the ATR kinase enhances 5-FU sensitivity independently of nonhomologous end-joining and homologous recombination repair pathways. J Biol Chem. 2020;295(37):12946–61. 10.1074/jbc.RA120.013726. PMC7489910 32675286

[40] Chun KS, Joo SH. Modulation of reactive oxygen species to overcome 5-fluorouracil resistance. Biomol Ther (Seoul). 2022;30(6):479–89. 10.4062/biomolther.2022.017. PMC9622305 35440517

[41] Lee S, Lee J, Lee GS, Moon JH, Jung J. Exploratory proteomic profiling reveals potential mediators of 5-FU response under p53 deficiency in colon cancer cells. Biomol Ther (Seoul). 2026;34(2):391–400. 10.4062/biomolther.2024.234. PMC12961981 41755778

[42] Carotenuto P, Pecoraro A, Brignola C, Barbato A, Franco B, Longobardi G, et al. Combining β-carotene with 5-FU via polymeric nanoparticles as a novel therapeutic strategy to overcome uL3-mediated chemoresistance in p53-deleted colorectal cancer cells. Mol Pharm. 2023;20(5):2326–40. 10.1021/acs.molpharmaceut.2c00876. PMC10155186 36976623

[43] Zhao H, Liu Q, Wang S, Dai F, Cheng X, Cheng X, et al. In vitro additive antitumor effects of dimethoxycurcumin and 5-fluorouracil in colon cancer cells. Cancer Med. 2017;6(7):1698–706. 10.1002/cam4.1114. PMC5504307 28573788

[44] Ong SKL, Shanmugam MK, Fan L, Fraser SE, Arfuso F, Ahn KS, et al. Focus on formononetin: anticancer potential and molecular targets. Cancers (Basel). 2019. 10.3390/cancers11050611. PMC6562434 31052435

